# Functional metal–organic liquids

**DOI:** 10.1039/d4sc01793e

**Published:** 2024-05-08

**Authors:** Nattapol Ma, Soracha Kosasang, Ellan K. Berdichevsky, Taichi Nishiguchi, Satoshi Horike

**Affiliations:** a International Center for Young Scientists (ICYS), National Institute for Materials Science 1-1 Namiki Tsukuba Ibaraki 305-0044 Japan ma.nattapol@nims.go.jp; b Centre for Membrane Separations, Adsorption, Catalysis and Spectroscopy (cMACS), KU Leuven Celestijnenlaan 200F 3001 Leuven Belgium; c Department of Chemistry, Graduate School of Science, Kyoto University Kitashirakawa-Oiwake-cho, Sakyo-ku Kyoto 606-8502 Japan horike.satoshi.3r@kyoto-u.ac.jp; d Department of Synthetic Chemistry and Biological Chemistry, Graduate School of Engineering, Kyoto University Katsura, Nishikyo-ku Kyoto 615-8510 Japan; e Institute for Integrated Cell-Material Sciences, Institute for Advanced Study, Kyoto University Yoshida-Honmachi, Sakyo-ku Kyoto 606-8501 Japan; f Department of Materials Science and Engineering, School of Molecular Science and Engineering, Vidyasirimedhi Institute of Science and Technology Rayong 21210 Thailand

## Abstract

For decades, the study of coordination polymers (CPs) and metal–organic frameworks (MOFs) has been limited primarily to their behavior as crystalline solids. In recent years, there has been increasing evidence that they can undergo reversible crystal-to-liquid transitions. However, their “liquid” states have primarily been considered intermediate states, and their diverse properties and applications of the liquid itself have been overlooked. As we learn from organic polymers, ceramics, and metals, understanding the structures and properties of liquid states is essential for exploring new properties and functions that are not achievable in their crystalline state. This review presents state-of-the-art research on the liquid states of CPs and MOFs while discussing the fundamental concepts involved in controlling them. We consider the different types of crystal-to-liquid transitions found in CPs and MOFs while extending the interpretation toward other functional metal–organic liquids, such as metal-containing ionic liquids and porous liquids, and try to suggest the unique features of CP/MOF liquids. We highlight their potential applications and present an outlook for future opportunities.

## Abstract 2

This review explores principles governing melting behaviors in coordination polymers and metal–organic frameworks, overviews their practical applications, and discusses design strategies for achieving liquid states in diverse metal–organic liquids.

## Graphical abstract

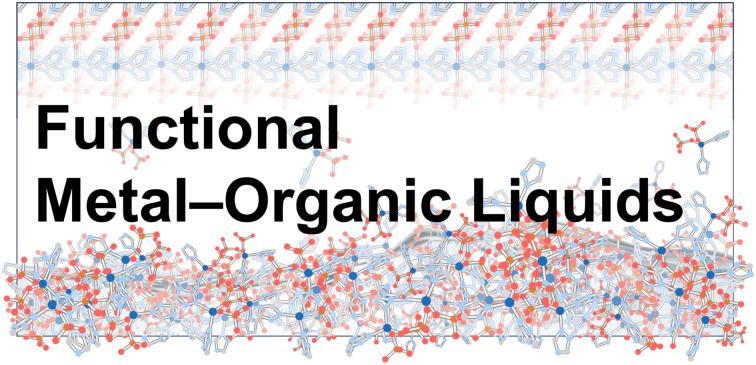

## 1. Introduction

**Fig. 1:**
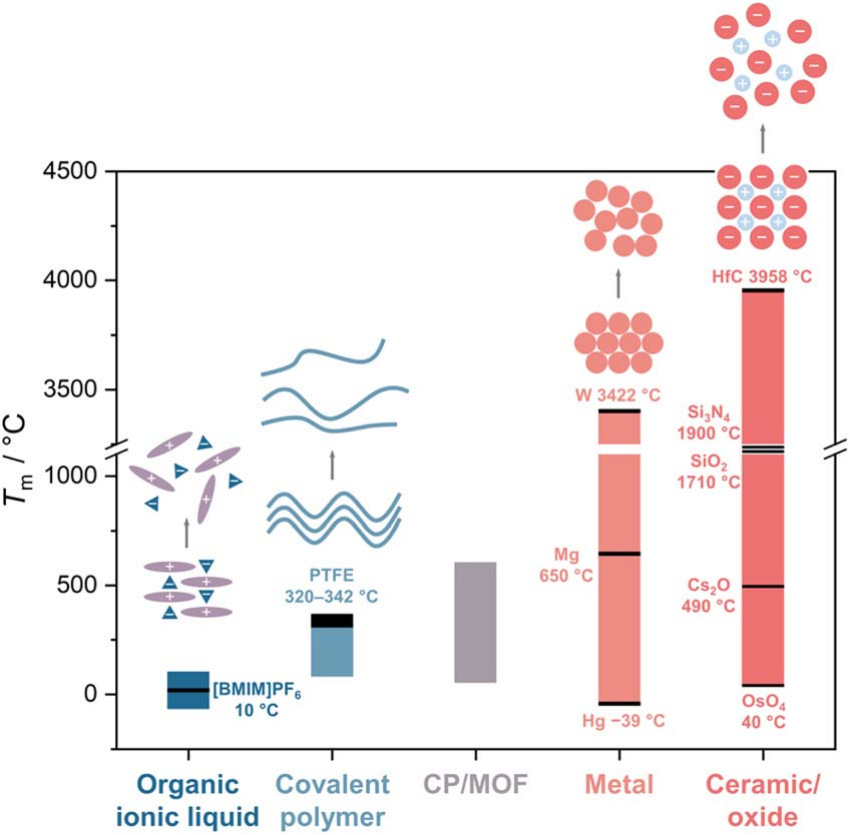
Approximate melting temperature range in organic ionic liquid, covalent polymer, CP/MOF, metal, and ceramic/oxide.

Solid-to-liquid transition, a ubiquitous yet complex phenomenon, is often described as one of the most fundamental and pivotal processes in materials science.^1^ It offers opportunities for material processing and the realization of exotic functionalities. For example, the melt growth process from their liquid states allows the production of large single crystals of Si and LiNbO_3_, essential materials in semiconductor technology and ferroelectric applications.^2–4^ The quenching of liquid states also yields glass, a transformation that is significant in optical and materials engineering.^5^ Accessing melting involves the strategic loosening of cohesive forces at melting temperature (*T*_m_, Fig. 1). For example, Ga melts at low *T*_m_ due to its large atomic size (weak metallic bond) and unusual crystal structure.^6^ For organic polymers, factors like molecular weight, degree of cross-linking, and composition all play pivotal roles in controlling the *T*_m_.^7^ Reducing the network size in silica glass decreases the processing temperature (Fig. 6).^8^ The reduction of *T*_m_ in ionic liquids is predominantly driven by entropy changes.^9^ These examples highlight diverse mechanisms involved in controlling melting behavior across different material classes and opportunities within this solid–liquid transition in materials science.

Coordination polymers (CPs) and metal–organic frameworks (MOFs) are solid materials with repeating coordination entities.^10–14^ They are assembled from metal nodes, commonly d- or f-block metal cations or clusters (secondary building units, SBUs), and linkers capable of bridging metal nodes *via* coordination bonds to generate polymeric arrays extended in one, two, or three dimensions.^15^ Robust and predictable coordination bonds enable the precise positioning of atoms in three-dimensional (3D) space, while the presence of organic linkers allows chemists to pre-program functional side groups. These important features of CPs/MOFs allow the control of structures and properties to be feasible even at the molecular level.^12,13^ Their functions have covered a wide range of fields, such as gas separation and storage, catalysis, optics, charge and mass transport, magnetics, and more.^16–27^ However, the development progress has predominantly revolved around the crystalline phase.

**Fig. 2:**
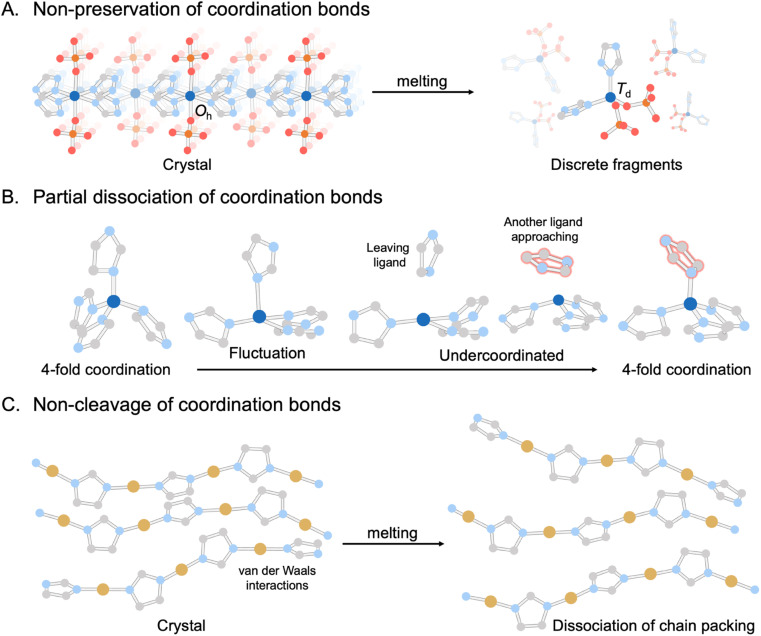
Three main categories of melting behaviors found in CPs/MOFs: (A) non-preservation of coordination bonds or ionic liquid-like structures, represented by Zn(H_2_PO_4_)_2_(HTr)_2_ (HTr = 1,2,4-triazole).^28^ (B) Partial dissociation of coordination bonds, represented by ZIF-4.^29^ (C) Non-cleavage of coordination bonds as a polymer-forming liquid, represented by Cu(2-isopropylimidazolate).^30^ Zn, C, N, O, P, and Cu atoms are represented by dark blue, gray, light blue, red, orange, and brown, respectively. Isopropyl groups and H atoms are omitted for clarity.

**Fig. 3:**
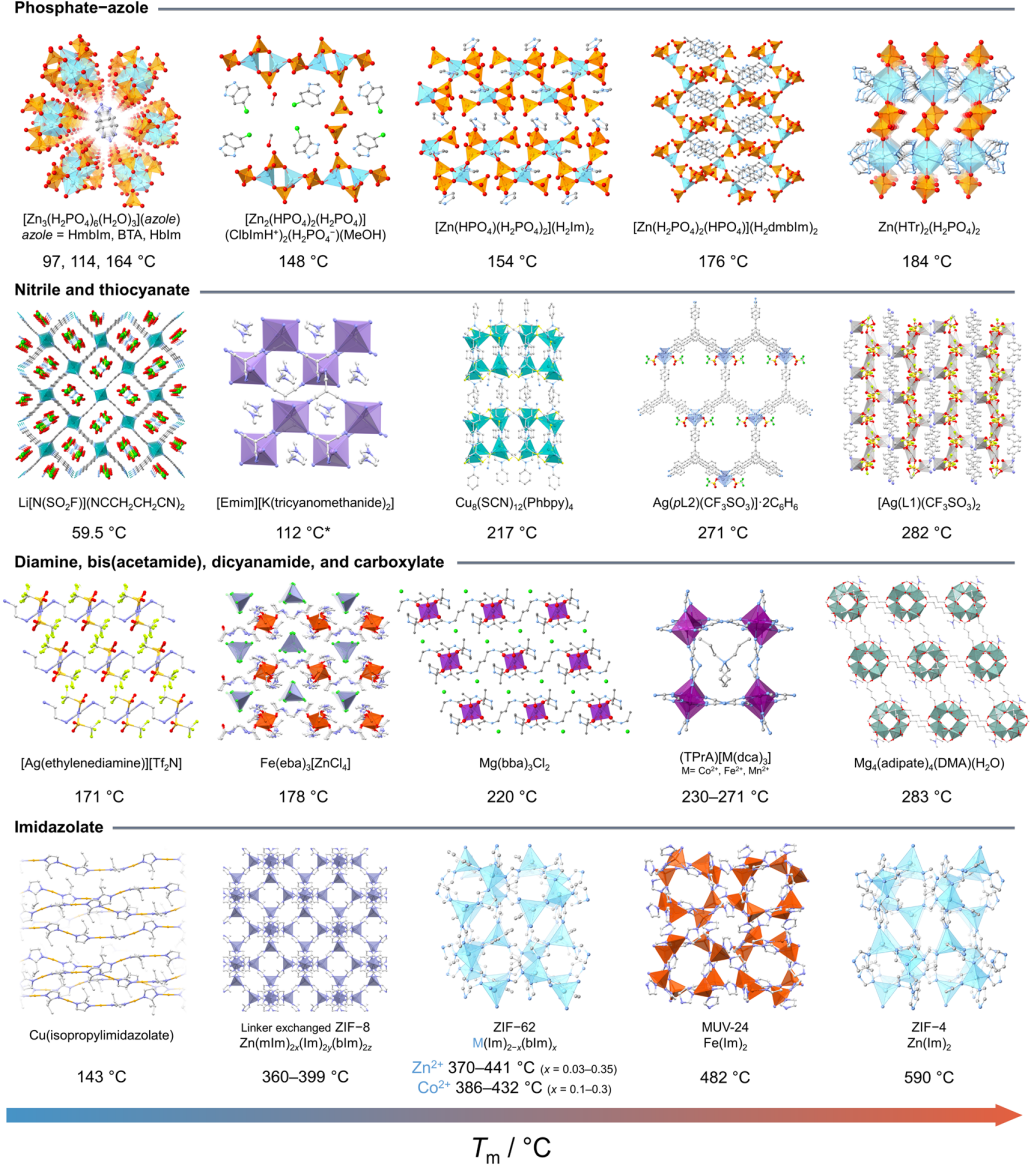
Examples of CPs and MOFs known to melt and their melting temperature (*T*_m_). Phosphate-azole from left to right: [Zn_3_(H_2_PO_4_)_6_(H_2_O)_3_](azole) (azole = HmbIm = 2-methylbenzimidazole,^31^ BTA = benzotriazole,^32^ HbIm = benzimidazole).^31^ [Zn_2_(HPO_4_)_2_(H_2_PO_4_)](ClbImH^+^)_2_(H_2_PO^4−^)(MeOH) (ClbIm = 5-chloro-1*H*-benzimidazole).^33^ [Zn(HPO_4_)(H_2_PO_4_)_2_](H_2_Im)_2_ (HIm = imidazole).^34^ [Zn(H_2_PO_4_)_2_(HPO_4_)](H_2_dmbIm)_2_ (HdmbIm = 5,6-dimethylbenzimidazole).^35,36^ Zn(HTr)_2_(H_2_PO_4_)_2_ (HTr = 1,2,4-triazolate).^31^ Nitrile and thiocyanate: Li[N(SO_2_F)](NCCH_2_CH_2_CN)_2_.^37^ [Emim][K(tricyanomethanide)_2_] (Emim = 1-ethyl-3-methylimidazolium).^38,39^ Cu_8_(SCN)_12_(Phbpy)_4_ (Phbpy = 1-phenyl-[4,4′-bipyridin]-1-ium).^40^ [Ag(*p*L2)(CF_3_SO_3_)]·2C_6_H_6_ (*p*L2 = 1,3,5-tris(4-cyanophenylethynyl)benzene).^41^ Ag(L1)(CF_3_SO_3_)_2_ (L1 = 4,4′-biphenyldicarbonitrile).^42^ Diamine, bis(acetamide), dicyanamide, and carboxylate: [Ag(ethylenediamine)][Tf_2_N].^43^ Fe(eba)_3_[ZnCl_4_] (eba = ethylenebis(acetamide)).^44^ Mg(bba)_3_Cl_2_ (bba = butylenebis(acetamide)).^45^ (TPrA)[M(dca)_3_] (TPrA = tetrapropylammonium, dca = dicyanamide).^46,47^ Mg_4_(adipate)_4_(DMA)(H_2_O).^48^ Imidazolate: Cu(isopropylimidazolate).^30,49^ Linker exchanged ZIF-8 (Zn(mIm)_2*x*_(Im)_2*y*_(mbIm)_2*z*_, (*x* + *y* + *z* = 1), mIm = methylimidazolate, Im = imidazolate, bIm = benzimidazolate).^50^ ZIF-62 (M(Im)_2−*x*_(bIm)_*x*_, M = Zn^2+^ or Co^2+^).^51,52^ MUV-24 (Fe(Im)_2_).^53^ ZIF-4 (Zn(Im)_2_).^54^ H are omitted for clarity.

In recent years, the concept of multistability within crystalline CPs/MOFs has expanded beyond the transformation between two stable crystalline states, observed in soft porous crystals,^55–66^ to further include the complete structural transformations in stable liquid and glass states.^31,54,67^ The emergence of liquid and glass CPs/MOFs presents opportunities for material processing and the exploration of novel or improved features inherent to the original crystals. Although melting and vitrification are common in many material families, they remain exotic within the context of CPs/MOFs since most tend to decompose upon heating due to the irreversible decomposition prior to reaching the *T*_m_. This led to the exploration of alternative methods that circumvent the need for melting, including mechanical vitrification,^68–72^ direct synthesis,^73,74^ and dehydration.^75,76^ Since the first observation of stable liquid states^34^ and vitrification,^31,54,67^ examples of CPs/MOFs exhibiting crystal melting have recently expanded beyond d^10^ metal ions to include a broader range of transition metals and a more comprehensive selection of linkers (Fig. 2 and 3).^44,45,48,53,77,78^

Before getting into the details, it is crucial to establish a clear understanding of the term “metal–organic liquid” and describe the scope of this review. Broadly, this term encompasses liquid materials comprising metal ions and organic components that link into repeating entities under certain states. The term includes a diverse array of entities known to exhibit a liquid state, ranging from metallo-supramolecular polymers,^79–82^ metal–organic cages/polyhedra,^83–86^ metal complexes,^87–89^ and certain metal-containing ionic liquids,^88,90–96^ among others. Many were known before the observation of melting behavior in CPs/MOFs. The distinctions between these systems become blurred upon the transition from solid to liquid. For instance, while CPs/MOFs exhibit extended coordination bonding networks in their crystalline state, such characteristics may not persist upon melting. The behavior of CPs/MOFs upon melting varies across individual systems; some undergo complete dissociation of coordination bonds, resembling discrete metal complexes in their liquid state,^28^ while others exhibit viscoelastic profiles similar to ionic liquids^31^ or retain polymeric characteristics with coordination bonds intact across all states.^30^ This review predominantly discusses the fundamental concepts and recent advancements in the design of stable liquid states for CPs/MOFs, particularly highlighting their melting behavior, structures, and properties. Our focus is on the systems that maintain well-defined, extending coordination bonding networks in specific states. Additionally, strategies for achieving thermodynamically stable liquid states are discussed, including insights from neighboring systems such as metal-containing ionic liquids, which possess well-defined structures but melt below room temperature, and porous liquids.

## 2. Metal–organic liquids and supercooled liquids

### 2.1 Thermodynamics of phase change and melting temperature

**Fig. 4:**
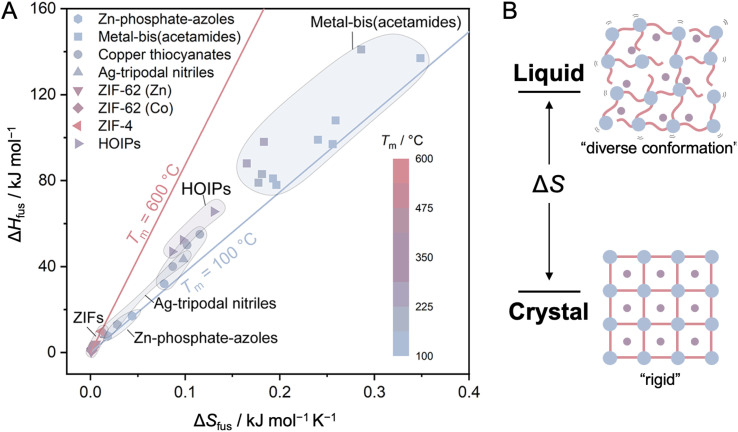
(A) Comparison of the difference in enthalpy of fusion (Δ*H*_fus_), the difference in entropy of fusion (Δ*S*_fus_) between solid and liquid phases, and the melting temperature (*T*_m_) of selected CP/MOF glasses. Each symbol shape denotes a series of compounds, while the symbol color represents *T*_m_. Data are taken from the following references: Zn–phosphate-azole,^28,31,32,34^ metal-bis(acetamides),^45^ copper thiocyanates,^40^ Ag-tripodal nitriles,^41,69^ ZIF-62,^52,97,98^ ZIF-4,^54^ and HOIPs.^47^ Adapted with permission from ref. 78. Copyright 2022 American Chemical Society under Creative Commons license CC-BY-NC-ND 4.0. https://creativecommons.org/licenses/by/4.0/. (B) Difference in entropy (Δ*S*) upon crystal-to-liquid transition.

Stabilizing a liquid state involves balancing melting (*T*_m_) and decomposition (*T*_d_) temperatures, usually under atmospheric pressure, which is achieved by either lowering *T*_m_ or raising *T*_d_. Melting is understood as the transition of particles between two or more equilibrium phases at a well-defined temperature and pressure and is described in the context of chemical potential (*μ*). *μ* is defined as the partial deviation of *G* with respect to the amount of substance (*n*): *μ* = (∂*G*/∂*n*)_*P*,*T*_. The differential of Gibbs energy is written as d*G* = *V*d*P* − *S*d*T* + *μ*d*n*. The differential of Gibbs energy is also given by d*G* = *n*d*μ* + *μ*d*n*. Hence, *n*d*μ* = *V*d*P* − *S*d*T* is derived. This equation is also expressed using molar volume *V*_m_ and molar entropy *S*_m_: d*μ* = *V*_m_d*P* − *S*_m_d*T* and is known as the Gibbs–Duhem equation. Since melting involves the migration of particles between solid and liquid phases in equilibrium, the deviation of the chemical potential of each phase is balanced as d*μ*_solid_ = d*μ*_liquid_. By employing the Gibbs–Duhem equation, the equation *V*_m,solid_d*P* − *S*_m,solid_d*T* = *V*_m,liquid_d*P* − *S*_m,liquid_d*T* is obtained. Transition entropy Δ_tr_*S*_m_ and transition volume Δ_tr_*V*_m_ are defined as the difference of entropy and volume of each phase, and d*P*/d*T* = Δ_tr_*S*_m_/Δ_tr_*V*_m_ is derived. The *T*_m_ is defined as the ratio of melting enthalpy and melting entropy: *T*_m_ = Δ*H*_fus_/Δ*S*_fus_. Therefore, *T*_m_ is minimized by minimizing the Δ*H*_fus_ while maximizing the Δ*S*_fus_ (Fig. 4).

Minimizing Δ*H*_fus_ involves minimizing cohesive chemical interactions among the components, including coordination bonds, hydrogen bonds, electrostatic interactions, and van der Waals interactions. Upon melting, the system should allow a certain degree of dissociation, which later translates into micro fluctuation and macroscopic fluidity in the liquid state (Fig. 2). This involves total coordination bond dissociation,^28,31^ partial coordination bond dissociation,^29^ or the dissociation of van der Waals interactions while maintaining the coordination bond.^30^ For example, pairing a d^10^ transition metal ion with lower crystal field stabilization energy with organic linker forming of weak coordination bonds would indeed require comparatively lower energy to dissociate the coordination bond. On the other side, the entropy of extended systems is interpreted as the summation of rotational, vibrational, and configurational terms, ignoring electronic or spin states. Δ*S*_fus_ reflects the freedom of structure and mobility. Designing CPs/MOFs with low-symmetry, high-flexibility linkers in which mobility is restricted in the crystalline state should give rise to the term Δ*S*_fus_ since diverse conformations are only accessible upon melting. Chemical component paring and resulting crystal structures influence these thermodynamic terms. Weak coordination bonds between cations and ligands, as well as weak electrostatic interactions between cations and anions, together minimize Δ*H*_fus_. The conformational flexibility of the bridging ligands also helps maximize Δ*S*_fus_.

The next question is: which of enthalpy or entropy contributes more to lowering the *T*_m_? To investigate this, we turn our attention to a study that delves into the origins of *T*_m_ in ionic liquids (ILs).^9^ Comparing Δ*H*_fus_ and Δ*S*_fus_ of 20 alkali halides and 257 ILs shows that Δ*S*_fus_ plays a more critical role (2.67 times larger in ILs) compared to Δ*H*_fus_ (0.85 times larger) on *T*_m_ lowering. Even in the case where conformational entropy in the cation and the anion is absent in ILs, a larger Δ*S*_fus_ still contributes more to reducing the *T*_m_. To further quantify the origin of the large entropic contribution, the Δ*S*_fus_ of imidazolium-based ionic liquids (imILs) was decomposed into kinetic (*S*_kin_) and structural (*S*_str_) entropies. The former includes translational (*S*_tra_), rotational (*S*_rot_), and intramolecular vibration (*S*_vib_). The latter comprises conformational or intramolecular (*S*_conf_) and configurational or intermolecular (*S*_config_) entropies. The Δ*S*_kin_ of imILs is smaller than NaCl, with Δ*S*_vib_ remaining close to zero or even showing a negative value. This behavior is attributed to the lack of significant changes in intramolecular vibration during the melting process and the presence of inactive diffusive motions resulting from the lower *T*_m_ of ILs. A large Δ*S*_str_, especially driven by a considerable Δ*S*_config_, significantly contributes to the reduction of *T*_m_. The origin of Δ*S*_config_ is associated with the existence of multiple configurations, characterized by the presence of delocalized charges and an asymmetric ion structure (Fig. 4B).

The elongation of alkyl chains on the cation of ILs is directly associated with an increase in Δ*S*_conf_ and is concurrent with an elevation in Δ*S*_fus_, while having minimal impact on both Δ*S*_config_ and Δ*S*_kin_.^99^ This behavior is illustrated in 1-alkyl-3-methylimidazolium bis(trifluoromethylsulfonyl)amide ([C_*n*_mim][NTf_2_], where *n* = 2, 4, 6, 8, and 10). When comparing the molecular dynamics (MD) simulations between liquid and gas states, it was also found that the population of *trans* conformations for the cation is more preferred in liquid than that in gas. The higher stability of *trans* conformers is due to (1) van der Waals interactions between alkyl chains and (2) coulombic interactions between the cations and anions that are not disturbed in these conformers. The populations of trans conformers for anion are, on the other hand, almost identical for both states. Apart from introducing linear *n*-alkyl chains, the introduction of branched alkyl substituents simulates a reduction in the melting point (*T*_m_) of C_60_ derivatives.^100,101^ This is attributable to the presence of additional bulky, flexible groups, which contribute to an increase in Δ*S*_fus_. Simultaneously, the introduction of these groups disturbs the π–π interactions between the π-conjugated cores, leading to a decrease in Δ*H*_fus_.

Lindemann's rule is another important parameter describing the origin of melting behavior in crystalline solids.^102^ It predicts that melting occurs when the root mean square displacement of particles due to thermal vibration exceeds a certain percentage of the interparticle spacing.^103–105^ As a function of temperature, a measure of the stability of coordination bonds, or the Lindermann ratio, is quantified by the variance in metal–ligand thermal vibration (*u*) compared to the inter-atomic distance *d*.^31,102^*u* is the square root of the Debye–Waller factor and is obtained directly from a single crystal XRD. The coordination bond is considered unstable at a critical Lindemann ratio (*f* = *u*/*d*) of around 0.1 but is varied depending on structure and bonding.

**Fig. 5:**
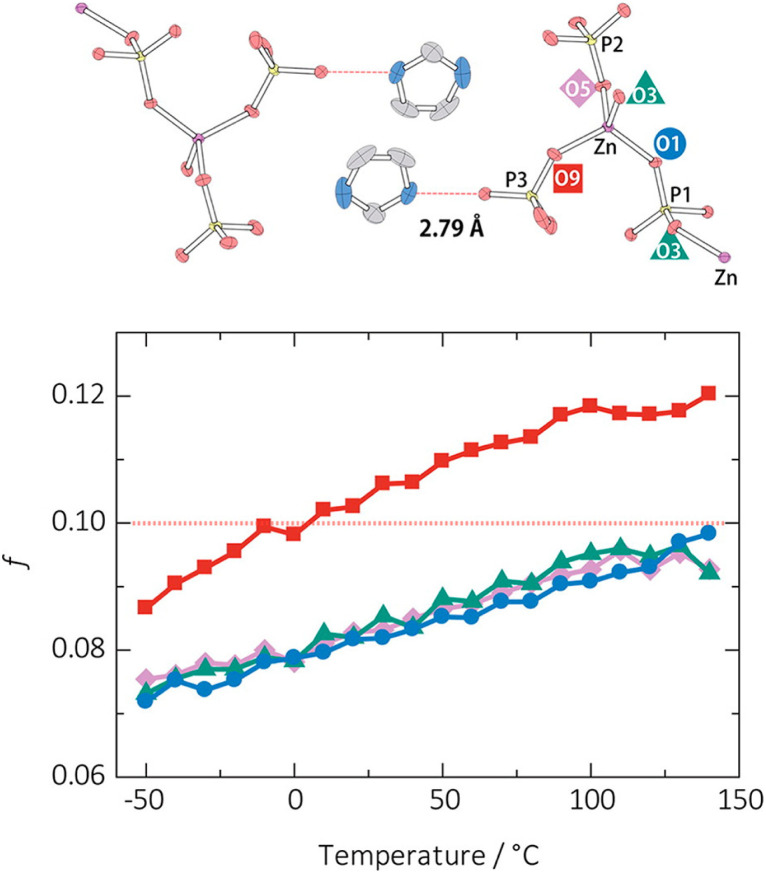
Degrees of thermal vibration of oxygen atoms around the Zn^2+^. O1 (blue circles), O3 (green triangles), O5 (purple diamonds), and O9 (red squares) and the ORTEP model and the H-bond distances of [Zn(HPO_4_)(H_2_PO_4_)](H_2_Im)_2_ (ZnPIm) at −50 °C. The Zn, P, O, N, and C atoms are shown in purple, yellow, red, blue, and gray, respectively; H atoms are omitted. Adapted with permission from ref. 31. Copyright 2015 American Chemical Society.

The determination of the Lindemann ratio is beneficial to clarifying the melting mechanism. An example illustrated in one-dimensional (1D) CP [Zn(HPO_4_)(H_2_PO_4_)_2_]·2H_2_Im (referred to as ZnPIm).^31^ The melting behavior is hypothesized to involve coordination bond dissociation within the 1D zinc–phosphate, ion-pair formation, and stabilization by its high ionicity. To clarify the melting event, the thermal vibration of oxygen atoms that coordinate with Zn^2+^ is analyzed (Fig. 5). Measuring single-crystal XRD under variable temperatures shows that the *f* value of all O atoms approaches 0.1 to 0.13 near *T*_m_. The coordinated oxygen atom of the H_2_PO_4_ ligand labeled as O9 exhibits a higher degree of thermal vibration than the other coordinated oxygen atoms (O1, O3, and O5). The H_2_PO_4_-contained O9 interacts with the imidazolium cation *via* hydrogen bonding, in which the imidazolium cation is rotatable at high temperatures, leading to its higher *f* value. The *f* value of O9 is 0.12, while O1, O3, and O5 are less than 0.10 at 140 °C, just below *T*_m_, suggesting that the melting event starts from the bond dissociation between Zn^2+^ and O9. The other Zn–O bonds dissociate after breaking the stable tetrahedral arrangement of the Zn^2+^ ion. The zinc–phosphate chains' loosening is induced by the mobility of imidazolium cations.

While most CPs/MOFs experience a complete transition from a crystalline solid to a liquid at a single temperature, referred to as congruent melting, some exhibit multistep transitions (incongruent melting), leading to the formation of a solid–liquid mixture (Table 1).^38,39,95,107^ In such cases, the temperature at which the compound begins to melt is termed the solidus temperature. The point at which complete liquefaction is achieved is referred to as the liquidus temperature. A compound melts congruently when its composition in the liquid state matches its original solid state. Some compounds become unstable during the transformation into a liquid, leading them to melt incongruently into their components instead of retaining their original composition.

**Table 1:** Selected CPs/MOFs systems showing incongruent melting behavior^a^

Materials	Note	*T*_solidus_/°C	*T*_liquidus_/°C	Ref.
[Ru(Cp)(C_6_H_5_C_2_H_5_)][Rb{C(CN)_3_}_2_]	3D	102.9	220	95
[Ru(Cp)(C_6_H_5_C_2_H_5_)][K{C(CN)_3_}_2_]	3D	132.9	—	95
[Ru(Cp)(C_6_H_5_CH_3_)][K{C(CN)_3_}_2_]	3D	214.6	—	95
[Emim][K(TCM)_2_]	2D	112.3	240	38 and 39
[EtPy][K(TCM)_2_]	2D	107.7	—	39
[PrPy][K(TCM)_2_]	2D	89.9	241	39
[N(C_4_H_8_)_2_][K(TCM)_2_]	2D	191.6	233	39
[SEt_3_][K(TCM)_2_]	2D	73.3	—	39
[NEt_4_][K(TCM)_2_]	3D	130.6	—	39
[CsHSO_4_]_*x*_[ZnPIm]_1−*x*_	Binary system	71.3–85.8	82.5–155	107

a Emim^+^, Etpy^+^, PrPy^+^, N(C_4_H_8_)_2_^+^, SEt_3_^+^, and NEt_4_^+^ are 1-ethyl-3-methyl-imidazolium, 1-ethylpyridinium, 1-propylpyridinium, 5-azaspiro[4.4]nonan-5-ium, triethylsulfo-nium, and tetraethylammonium, respectively. *x* represents the molar fraction of CsHSO_4_ in the binary system. Note that the *T*_liquidus_ of some systems is not reported.

CPs with a composition of [Ru(Cp)(C_6_H_5_R)][M{C(CN)_3_}_2_] (R = Me, Et; M = K, Rb; Cp = C_5_H_5_) melt incongruently, forming a mixture of solid M[C(CN)_3_] salts and ionic liquids [Ru(Cp)(C_6_H_5_R)][C(CN)_3_].^95^ This occurs due to the low solubility of M[C(CN)_3_] in the ionic liquids. The melt states of these compounds are relatively unstable and show partial decomposition of the liquid phase. Replacing organometallic cations with Emim^+^ (1-ethyl-3-methyl-imidazolium), a representative ionic liquid component, improves the thermal stability of liquid states while maintaining incongruent melting behavior.^38^ Melting of [Emim][K(TCM)_2_] (TCM = tricyanomethanide) begins at *ca.* 112 °C, accompanied by an immediate growth and deposition of microcrystals. XRD and Raman confirm the composition of the solid–liquid mixture to be K[TCM] microcrystals and [Emim][TCM] ionic liquid. A uniform liquid is observed after the temperature reaches 240 °C. Correlations between CPs with incongruent melting behavior and their constituents are generalized in a series of CPs synthesized from onium ionic liquids and K[TCM].^39^ The *T*_m_ of CPs was linearly correlated with the *T*_m_ of ILs. The cooling rate required for vitrification was correlated with the flexibility of cations, with higher flexibility resulting in easier vitrification abilities. In addition to these pure compounds, a binary system composed of CP and CsHSO_4_ also shows incongruent melting and eutectic behavior.^107^ Detailed discussions are provided in the next section.

**Fig. 6:**
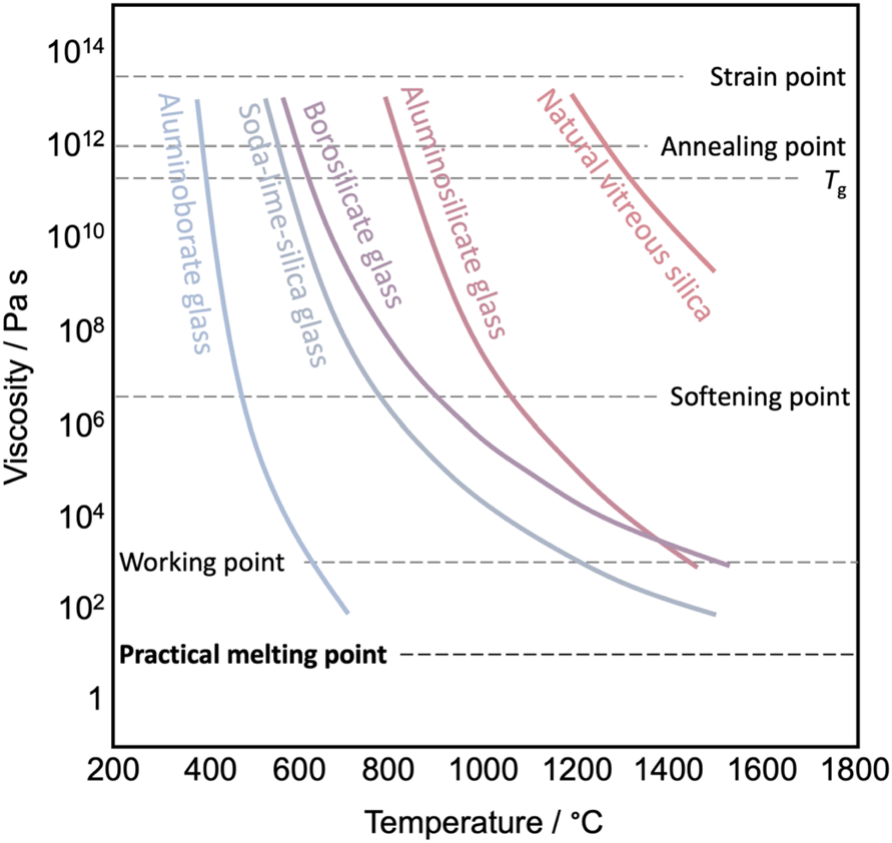
Viscosity *versus* temperature curve of various glasses and viscosity reference temperature. Data are taken from ref. 106.

The transition from a glassy state to a liquid state is more complex. The glass transition temperature (*T*_g_) marks the point at which there is a noticeable change in the temperature-dependent thermodynamic properties, shifting from values resembling those of a crystal to those of a liquid.^108^ Below the *T*_g_, materials exist in a glassy state. When heated above its *T*_g_ but still below the liquidus point, these glasses undergo a transformation from a rigid, amorphous solid to a viscoelastic, supercooled liquid state. In this state, the supercooled liquid displays a viscous response, making it suitable for shaping. Once it cools down again to below *T*_g_, the supercooled liquid structure freezes to a solid glassy state, and the shape is maintained. The viscosity at *T*_g_ is ≈ 10^12.5^ Pa s (Fig. 6). When observing the viscoelastic behavior using dynamic mechanical analysis, the point at which the loss modulus (*G*′′) reaches its maximum (relaxation temperature) signifies the onset of a supercooled liquid state. Heating the supercooled liquid above the liquidus point (often equivalent to *T*_m_) results in the transition to the liquid phase (viscosity < 10^1^ Pa s). This viscosity is suitable for the melt to provide good homogeneity within the melting container, and air pockets are removed by vacuum. Handling some liquids might require an inert atmosphere to avoid oxidation or degradation.

**Fig. 7:**
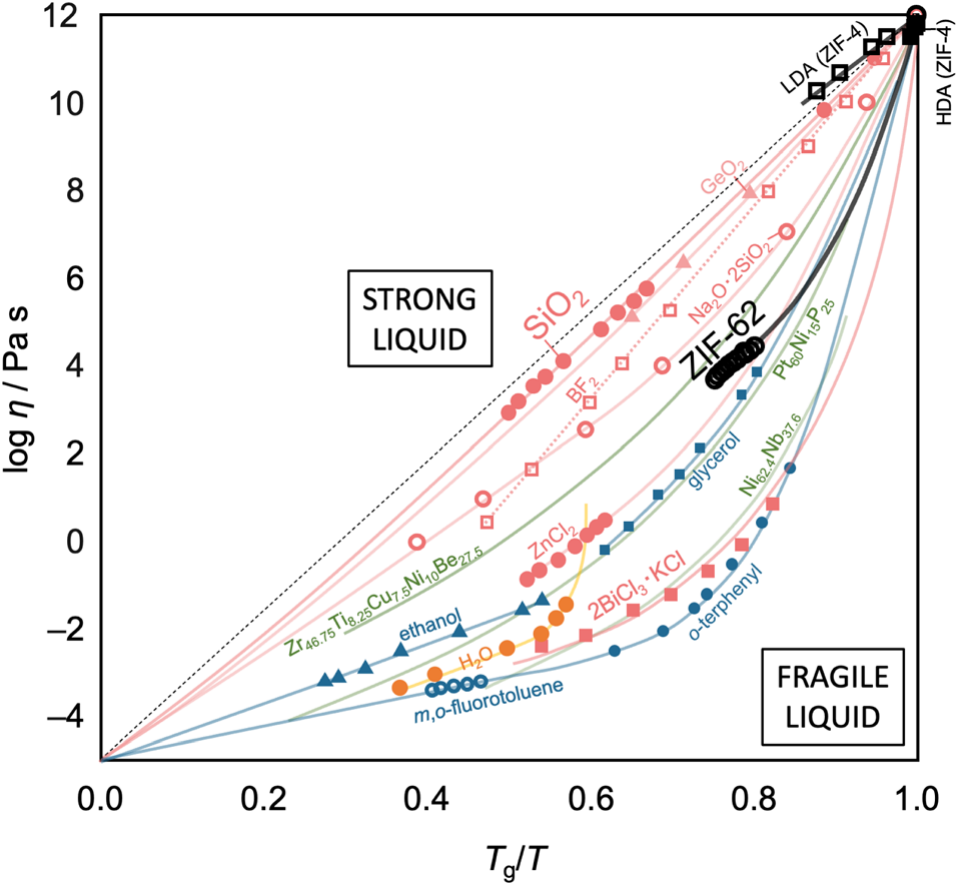
Fragility plot (Angell's plot) of selected glass-forming systems. Data are taken from ref. 5, 54, 97 and 113–115. HDA and LDA are the high-density and low-density amorphous phases of ZIF-4, respectively. Red, blue, green, orange, and black lines and symbols present inorganic, organic, metallic, water, and metal–organic systems, respectively.

For glass-forming liquids, the concept of liquid fragility has been used to classify the viscosity/temperature relations, which are represented by the fragility diagram (Fig. 7).^5,109,110^ A liquid is considered “strong” when it exhibits near-Arrhenian behavior over the entire viscosity range. In contrast, a liquid that displays a large degree of curvature is termed “fragile.” Generally, strong liquids maintain a high degree of short-range order and only allow minor dissociation of bonds upon increasing temperature.^111^ As a result, only a small change in heat capacity is observed when passing through the *T*_g_. On the other hand, fragile liquids tend to have less well-defined short-range order, and their structures disintegrate rapidly with temperatures increasing above *T*_g_ with a large change in heat capacity. In addition, the liquid fragility is usually quantified by the fragility index, *m*, where *m* = [d(log *η*)/d(*T*_g_/*T*)]_*T*=*T*_g__, which describes the slope of viscosity (*η*) with temperature as it approaches *T*_g_.^112^ For example, SiO_2_ has a *m* value of *ca.* 20 and is considered a strong liquid, while the *m* value of fragile liquids usually ranges between 40 and 50.

### 2.2 Melting behaviors and structures

Determining average structures in liquids and glasses is important for understanding their behavior and achieving functionality. It requires the integration of various techniques, such as the pair-distribution function (PDF), solid-state NMR, extended X-ray absorption fine structure (EXAFS), and atomistic modeling.^78^ The building blocks of CPs/MOFs, consisting of metal nodes and bridging linkers, establish interconnecting networks through coordination bonds in their crystalline state. When it is subjected to melting, these connections display diverse behaviors that fall into three main categories observed thus far (Fig. 2): (1) non-preservation of coordination bonds or ionic liquid-like structures, (2) partial dissociation of coordination bonds, and (3) non-cleavage of coordination bonds as a polymer-forming liquid.

The foremost behavior is observed in two-dimensional (2D) Zn(H_2_PO_4_)_2_(HTr)_2_ (HTr = 1,2,4-triazole) (Fig. 2A).^28^ The CP consists of octahedral (O_h_) Zn^2+^ with monocoordinated orthophosphate (axial position) and bridging 1,2,4-triazole, which form extended arrays of 2D sheets parallel to the *ab* plane. It melts at 184 °C. This initiates a local geometry transformation around Zn^2+^ from an octahedral (O_h_) in crystal to a tetrahedral (*T*_d_) arrangement in liquid. The change drives the structural transformation from 2D to 0D (discrete molecular fragment) and persists even after cooling in the glassy state. The emergence of these discrete molecular fragments highlights the similarity between the melting of CPs/MOFs and the behavior of ionic liquids. Conventionally, the network preservation between crystal and glass states in CP/MOF is rationalized by considering that the lattice enthalpies must be comparable in both states. This must be an exceptional case where the lattice enthalpies between the two states are comparable despite having very different structures.^116^

Slight variations in behavior are also observed in the case of 1D Zn–phosphate-azole: ZnPIm.^31^ A single sharp peak for the liquid state (160 °C) compared to the broad peak of glass and crystal displayed in the ^31^P solid-state NMR spectra confirms the discrete molecular fragments of Zn^2+^, phosphate, and imidazolium ions without coordination bond preservation, referring to an ionic liquid-like structure. A dynamic mechanical analysis (DMA) displays an immediately higher value of the loss modulus than the storage modulus (*G*′′ > *G*′) above 30 °C (*T*_g_), which is a typical profile of viscoelastic fluids. Suppose the coordination bonds are preserved upon melting, as in linear organic polymers. In that case, the effect of entangled chains should be presented as a rubbery plateau regime (*G*′ > *G*′′). This supports the cleaved-bond model without preserving coordination bonds. The viscosity of the liquid state, derived from the shear modulus, also follows the typical profile for ionic liquids. Despite the coordination bonds being cleaved and behaving like ionic liquids upon melting, PDF confirms that its 1D polymeric structure re-establishes upon cooling below the *T*_g_.

The melting process of Zn(Imidazolate)_2_ (ZIF-4) leads to the partial dissociation of coordination bonds (Fig. 2B).^29^ The compound consists of Zn^2+^ and imidazolate groups in a tetrahedral coordination arrangement, forming a 3D-crystalline network with a maximum cavity diameter of 4.9 Å. Note that ZIF-4 undergoes amorphization and recrystallization to become nonporous ZIF-zni upon heating before the melting process.^54^ Through first-principle molecular dynamics (FPMD) simulations, over 94% of Zn^2+^ maintains the ideal coordination number of 4, where Zn^2+^ coordinates with the N atom of four imidazole linkers just below the *T*_m_. The number of undercoordinations of Zn^2+^ in addition to 4-fold coordination above the *T*_m_, such as tri- and bi-coordination, arises due to the partial dissociation of Zn^2+^–N bonds. The snapshots of microscopic evolution during melting (FPMD at 1227 °C) reveal the breaking of Zn^2+^–N bonds and the reorientation of imidazolate linkers between adjacent coordination sites within picoseconds. Note that the higher temperature ranges in FPMD are not physically relevant for the experimental system but are necessary, due to the short time explored in FPMD, to gather statistics on relatively rare events and high thermodynamic barriers. A relatively small configurational difference between the crystalline state and its corresponding liquid translates into a small entropic difference when compared to other systems, together with a relatively low fragility index. The ZIF-4 melts also share a common feature with ionic liquids, where their constituents show comparable translational diffusion between imidazolate and Zn^2+^ of 7.7 × 10^−10^ and 6.5 × 10^−10^ m^2^ s^−1^, respectively.

An identical feature in melts has been observed in metal-bis(acetamide) frameworks, such as Co(*N*,*N*′-1,4-butylenebis(acetamide))_3_[CoCl_4_], in which the coordination number around Co^2+^ centers decreases by *ca.* 20% upon melting.^45^ In the molten state, the average coordination number is approximately 4.8, which significantly surpasses the bond percolation threshold of 2.4 required for a 3D aperiodic network. PDF analysis showcases the insignificant alteration of pair distances up to *ca.* 4 Å in the melt compared to the crystalline state and, more importantly, quasiperiodic oscillations extending up to 80 Å. The latter points to the existence of both topological and chemical ordering, thus verifying the partially retained extended-range order connectivity within the liquid.

The third type emerges when melting occurs without the dissociation of any bonds, resembling the melting characteristics of organic polymers (Fig. 2C). One example of a polymer-type forming liquid is found in 1D Cu(2-isopropylimidazolate) with a *T*_m_ of 143 °C.^30,49^ The initial crystal structure comprises 6.5 Cu^2+^-isopropylimidazolate units, each of which is crystallographically distinct. These 1D chains are assembled through van der Waals interactions. Heating above its *T*_m_, the compound maintains its coordination environment and intramolecular connectivity (N–Cu^+^–N). The main PDF peaks of the crystalline state (30 °C) at 6.1, 11.6, and 17.1 Å correspond to the nearest, second, and third neighbor correlations of two intrachain Cu^+^ ions. The peak at 6.1 Å remains mostly unchanged even above the *T*_m_, indicating the preservation of Cu^+^-isopropylimidazolate-Cu^+^ bridging within its liquid state. In contrast, the reduced intensity of peaks at 11.6 and 17.1 Å suggests lower structural periodicity. An insignificant change in coordination number from 2 in the crystalline state to 1.97 in the liquid state also supports the retention of the 1D chain structure model without bond breaking. ^1^H MAS solid-state NMR of the melt at 157 °C shows peaks narrowing due to higher molecular dynamics compared to the parent crystal. The retention of spinning side bands suggests the presence of weak anisotropic nuclear spin interactions between the ligands. A broader distribution of intermolecular Cu^+^–Cu^+^ correlation and the relatively high viscosity with storage modulus above the *T*_m_ suggest that the melting is due to the dissociation of chain packing and entangled networks of stiff chains rather than the dissociation of coordination bonds as observed in previous examples.

**Fig. 8:**
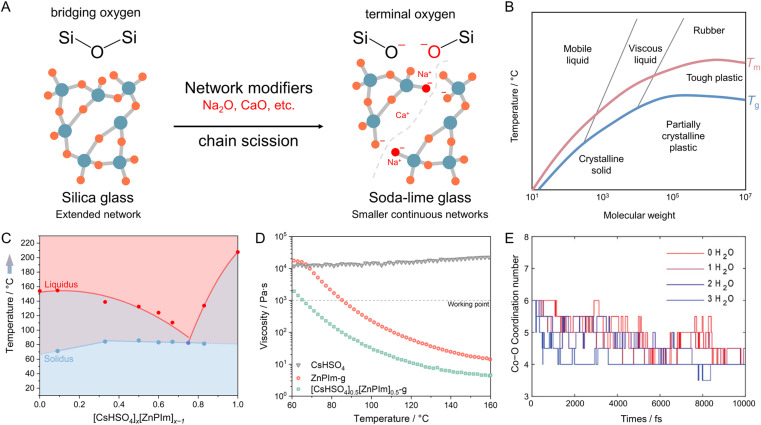
Modification of melting behavior and properties of melts through the addition of network modifiers. (A) Schematic diagram depicting the process of soda-lime silica glass formation by introducing network modifiers like Na_2_O and CaO, accompanied by their corresponding temperature-dependent viscosity profiles. (B) Approximate relation between molecular weight, glass transition temperature (*T*_g_, blue line), melting point (*T*_m_, red line), and polymer properties.^117^ (C) A diagram demonstrating composition-dependent solidus and liquidus points of the CsHSO_4_–ZnPIm binary system at ambient pressure. Blue hexagons and red circles represent the invariant point and temperature of the liquidus, respectively. The diagram has three regions: mixed solids (blue), incongruent melting (purple), and liquid phase (red). (D) Temperature-dependent viscosity of CsHSO_4_, ZnPIm-g, and [CsHSO_4_]_0.5_[ZnPIm]_0.5_-g over heating scan. (E) Simulated average coordination number of the Co–O correlation in Co(hmba)_3_[CoBr_4_] structures containing 0, 1, 2, or 3 water molecules. (B and C) are adapted with permission from ref. 107. Copyright 2022 American Chemical Society. (D) is adapted with permission from ref. 118. Copyright 2023 John Wiley & Sons, Inc. under Creative Commons license CC BY 4.0. https://creativecommons.org/licenses/by/4.0/.

Viscosity modulation of liquid CPs/MOFs through the incorporation of network modifiers has been a longstanding technique to regulate the processing temperature of glass (Fig. 6 and 8A).^111^ Specifically, pure silica glass (quartz glass) composed of SiO_2_ typically demands temperatures as high as 1800 °C to achieve a viscosity appropriate for processing, owing to the presence of extended Si–O–Si connectivity. The viscosity of the melts is reduced by adding network modifiers such as Na_2_O, CaO, or MgO. Introducing terminal oxygen to reduce network connectivity results in a lower viscosity (lower *T*_m_ and *T*_g_) and thus lower processing costs. The concept is also known as chain scissions, but it is less known for meltable CPs/MOFs. The same principle is also applicable for controlling the thermal behavior of polymers since their properties are dictated by molecular weight (Fig. 8B).^7^

A superionic solid acid, CsHSO_4_, functions as a network modifier when introduced to ZnPIm.^107^ As previously mentioned, ZnPIm consists of a 1D Zn–phosphate coordination chain and non-coordinated H_2_Im molecules. The physical mixing of ZnPIm (*T*_m_ = 154 °C) with CsHSO_4_ (*T*_m_ = 206 °C) leads to changes in the mixture's *T*_m_, revealing composition-dependent melting behavior (Fig. 8C). For example, the DSCs of the mixture [CsHSO_4_]_*x*_[ZnPIm]_1−*x*_ with equivalent mol fraction (*x* = 0.5) show first endothermic peaks with an onset temperature of 85.8 °C of crystal melting. The value is much lower for both constituents. The melting process remains incomplete, and a portion of the crystalline domain persists until it completely melts beyond 140 °C. This behavior is referred to as incongruent melting. The solidus point marks the temperature at which the mixture starts to melt (85.8 °C), while the liquidus point signifies the temperature at which the mixture achieves complete melting (140 °C). The behavior is also observable through variable-temperature XRD and SEM. At eutectic composition (*x* ≈ 0.75), only a single endothermic peak is observed, showing the lowest *T*_m_ and defining the eutectic point. This is described as the first example of eutectic melting observed in the system of CPs/MOFs. In addition to exhibiting eutectic behavior, cooling the melts back also results in a binary glass formation that helps preserve the superprotonic conductivity of CsHSO_4_ and will be later discussed in Section 4.1. The enhanced conductivity within the binary system arises from changes in their structure and the role of CsHSO_4_ as a network modifier for ZnPIm. ^31^P magic-angle spinning (MAS) solid-state NMR reveals a partial reorientation of bridging phosphate to a monodentate type due to the oxyanion exchanged. The tetrahedral coordination of Zn^2+^ is filled by HSO_4_^−^ from CsHSO_4_, leading to chain scission of the primary 1D chain of ZnPIm. This phenomenon reduces the overall viscosity (*η* < 10^3^ Pa s at 65 °C) of the system while simultaneously enhancing the molecular mobility within the system, resembling silica glass when network modifiers are introduced (Fig. 8D).

Water also acts as a network modifier to break the continuous network of a meltable Co(hmba)_3_[CoBr_4_], where hmba represents *N*,*N*′-1,6-hexamethylene-bis(acetamide).^118^ In the crystalline state, Co^2+^ nodes are coordinated in an octahedral manner to hmba, forming a 2D layered-like structure. Non-coordinate [CoBr4]^2−^ units are situated between these layers to balance the overall charge. By carefully controlling the addition of water, it's possible to induce alterations in both the *T*_m_ and *T*_g_ of the glass. The *T*_m_ decreases from 110 °C to ∼70 °C by adding ∼7 wt% water (∼4.6 water molecules per bridging Co^2+^), whereas the *T*_g_ is also lowered to −20 °C compared to 20 °C of the pristine compound. Note that the amount of added water is within the stability limit, and excessive water addition leads to the complete dissociation of the coordination network. Similar effects of decreasing *T*_m_ are also observed when extending toward 2D Mn(hmba)_3_[MnBr_4_] and 3D Co(hmba)_3_[Co(SCN)_4_]. Adding water also promotes the glass-forming ability of Mn(hmba)_3_[MnBr_4_] by suppressing the reformation of the initial network-connecting species. X-ray analyses conducted on anhydrous and hydrated Co(hmba)_3_[CoBr_4_] also indicate that the presence of water triggers partial decoordination of linkers by forming coordination bonds with Co^2+^ nodes, even before the *T*_m_ is reached. To further understand the mechanism, the mean square displacement (MSD) and coordination number (CN) of Co atoms were calculated *via ab initio* MD (Fig. 8E). At 727 °C, a temperature corresponding to the melting event (as indicated by the Lindemann ratio between 1.0–1.5), the Co MSD increases more rapidly with time in the presence of water. This suggests that water accelerates the melting process of Co(hmba)_3_[CoBr_4_]. This effect becomes even clearer when examining the change in CN. The results show that the presence of water induces a shift in the melting behavior by promoting the decoordination of hmba ligands. This leads to a lower *T*_m_. The impact of water is evident in the accelerated decrease of the CN only for the networking Co–O interactions, while the Co–Br interactions of the anion remain mostly unaffected. These mechanisms observed in the two examples here resemble those of network modifiers used in traditional oxide glass-forming systems to design and tune the liquid and glass properties.

Modification of structural composition leads to an accessible *T*_m_ in non-melt CP/MOFs. Unlike ZIF-4, the organic linker in Zn(2-methylimidazole)_2_ (ZIF-8) faces instability when the Zn^2+^–N bonds partially dissociate.^119^ This instability hinders ZIF-8 from undergoing a melting process and instead leads to its decomposition upon heating (*T*_m_ > *T*_d_ = 550 °C). This is due to a large bond cleavage activation energy difference and because the nearby coordination sites cannot promptly accommodate the departing linkers due to their low density. Stabilizing agents, such as ionic liquid, are an option to counteract this challenge and help stabilize the rapidly dissociating linkers.^120^ Incorporating 1-ethyl-3-methylimidazolium bis(trifluoromethanesulfonyl)imide ionic liquid, [EMIM][TFSI] (IL), into the pores of ZIF-8 (IL@ZIF-8) stabilizes the frameworks, resulting in an accessible *T*_m_ (below *T*_d_). The IL@ZIF-8 composite shows a small endothermic peak (*T*_m_) at 381 °C before decomposing at *ca.* 412 °C. The results suggest that electrostatic interaction between ZIF-8 and the partially decomposed IL fragments or IL itself helps stabilize the dissociating ZIF-8 components upon melting.

Structural transformation to a denser phase upon heating can lead to a meltable phase. ZIF-4 undergoes a multiple-phase transition, first to amorphous phases, and then transforms again to a denser zni topology before melting at 590 °C (*T*_d_ = 600 °C).^54^ A meltable Fe^2+^-based ZIF Fe(Im)_2_ is obtained from thermal treatment of Fe_3_(im)_6_(Him)_2_ at 283 °C to remove neutral imidazole.^53^ Further heating to 417 °C induces another transformation to denser zni phases before melting at 482 °C. The liquid state is stable until 550 °C. Although ZIF-4 with Co^2+^ shows identical transformation to the zni phase, further heating results in decomposition without melting.^52^

**Fig. 9:**
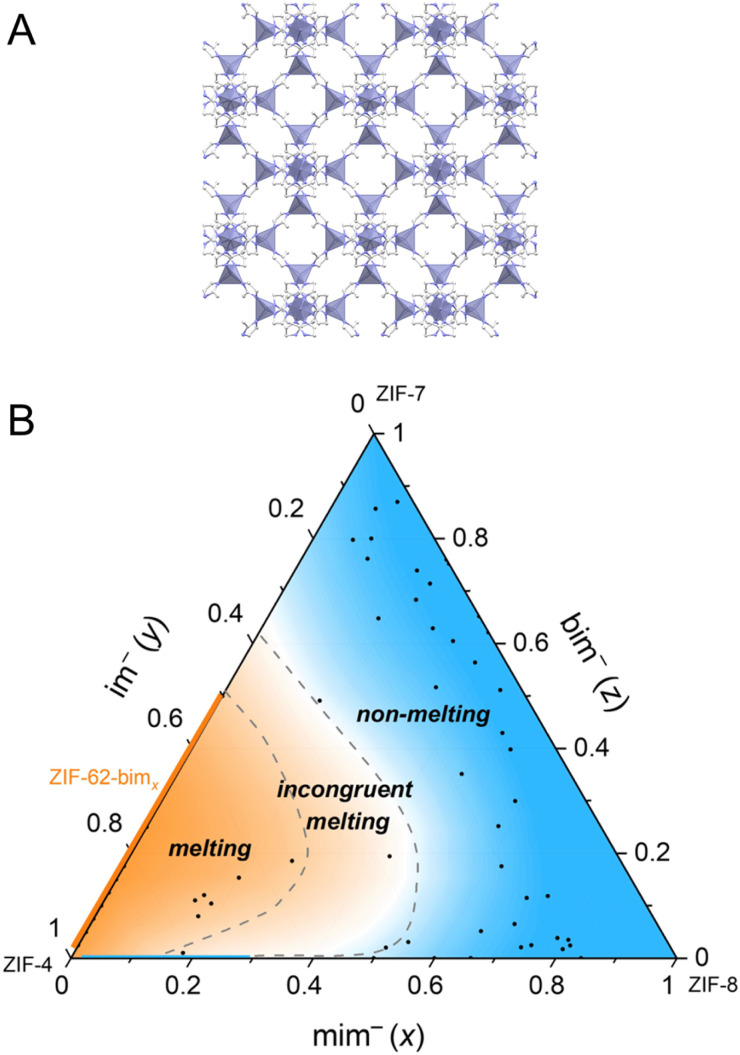
(A) Crystal structure of ZIF-8. Zn, C, and N atoms are presented in purple, grey, and blue. H atoms are omitted for clarity. (B) Ternary phase diagram based on thermal analysis and XRPD data of 50 derivates of ZIF-8-mim_*x*_im_*y*_bim_*z*_ along with literature data of ZIF-4 and ZIF-62-bim_*x*_ (orange line), with the blue area being the non-melting region, the area between the two dashed lines being the incongruent melting region, and the orange area being the melting region (excluding the blue line of ZIF-8-mim_*x*_im_*y*_). Adapted from ref. 50 under Creative Commons license CC BY 4.0. https://creativecommons.org/licenses/by/4.0/.

Another approach to creating a meltable version of ZIF-8 (Fig. 9A) involves a process called solvent-assisted linker exchange (SALE) with the incorporation of two additional organic linkers.^50^ In this method, partial exchange of the 2-methylimidazole linker (mim^−^) with a smaller and weakly coordinating imidazolate linker (im^−^) helps facilitate the bond-breaking process. Simultaneously, introducing a larger benzimidazolate (bim^−^) linker stabilizes the resulting melts while preventing the dense ZIF from crystallizing. By varying the molar ratios of these three linkers, a range of ZIF-8-mim_*x*_im_*y*_bim_*z*_ derivatives are produced. Some of these derivatives retain the same sod topology as ZIF-8. The inclusion of im^−^ alone leads to the formation of a small amount of zni phase (relating to ZIF-61).^121^ The introduction of bim^−^ into the mix reduces the occurrence of ZIF-61-like structures. As the bim^−^ ratio increases beyond 0.65, it results in the formation of either a cubic sod or a heavily distorted rhombohedral or triclinic sod structure. This composition-dependent behavior leads to a range of thermal characteristics, and the melting behavior is summarized in a ternary phase diagram, including the regions of congruent melting, incongruent melting, or no melting at all (Fig. 9B). Cooling of samples that melt congruently results in glass with *T*_g_ between 334 and 361 °C. On the other hand, samples showing incongruent melting provide crystal-glass composites after cooling consisting of crystalline ZIF-61 or ZIF-8.

### 2.3 Metal-containing ionic liquids

Metal-containing ionic liquids predate the discovery of melting behavior in CPs/MOFs, with some examples exhibiting extended coordination networks upon crystallization but melting at much lower temperatures. Considering the strong connection between CPs/MOFs in their molten state and metal-containing ionic liquids,^91^ delving into the design principles of the latter could aid us in better understanding the factors that govern the melting behavior of CPs/MOFs. One could also consider these metal-containing ionic liquids with well-defined structures as CPs with *T*_m_ below room temperature. In the first example presented here, the influence of hydrogen bonding on *T*_m_ has been examined in lanthanide-containing ionic liquid systems, such as [BMIM]_*x*−3_[Ln(NCS)_*x*_(H_2_O)_*y*_] (*x* = 6–8, *y* = 0–2, Ln = Y^2+^, La^3+^, Pr^3+^, Nd^3+^, Sm^3+^, Eu^3+^, Gd^3+^, Tb^3+^, Ho^3+^, Er^3+^, and Yb^3+^, with BMIM representing the 1-butyl-3-methylimidazolium cation).^90^ These systems predominantly exist as transparent or supercooled liquids at room temperature and tend to form glasses rather than crystals upon cooling. Selected few compounds with *x* = 6–8, *y* = 1–2, and Ln = La^3+^, Y^3+^, and Nd^3+^ were observed to crystallize at approximately 16 °C from the molten state, exhibiting *T*_m_ ranging from 28 °C (Nd^3+^) to 39 °C (Y^3+^). Among these compounds, the crystal structure of [BMIM]_4_[La(NCS)_7_(H_2_O)] was determined using single-crystal X-ray diffractometry. The coordination number of the Ln^3+^ ion is eight, including seven coordinated isothiocyanate anions and one coordinated water, where a slightly distorted square antiprism of the coordination polyhedron is observed. Each coordinated water molecule in [La(NCS)_7_(H_2_O)]^4−^ forms strong hydrogen bonds to the isothiocyanate anion of the neighboring unit with *d*(O–H⋯S) 2.48 and 2.58 Å, resulting in a columnar stacking of these units along the direction of the *a* axis. Acidic hydrogen atoms from four imidazolium cations surrounding each [La(NCS)_7_(H_2_O)]^4−^ moiety also form weak hydrogen bonds to the sulfur of the thiocyanate anions (C–H⋯S from 2.73 up to 2.84 Å). The crystallization ability of metal-containing ionic liquids is attributed to hydrogen bonding formed by coordinated water molecules that leads to the polymeric stacking of anions. In comparison, [BMIM]_5_[Ln(NCS)_8_] without O–H⋯S hydrogen bonding capability does not exhibit a solid state even at −20 °C, where it behaves as highly viscous liquids.

**Fig. 10:**
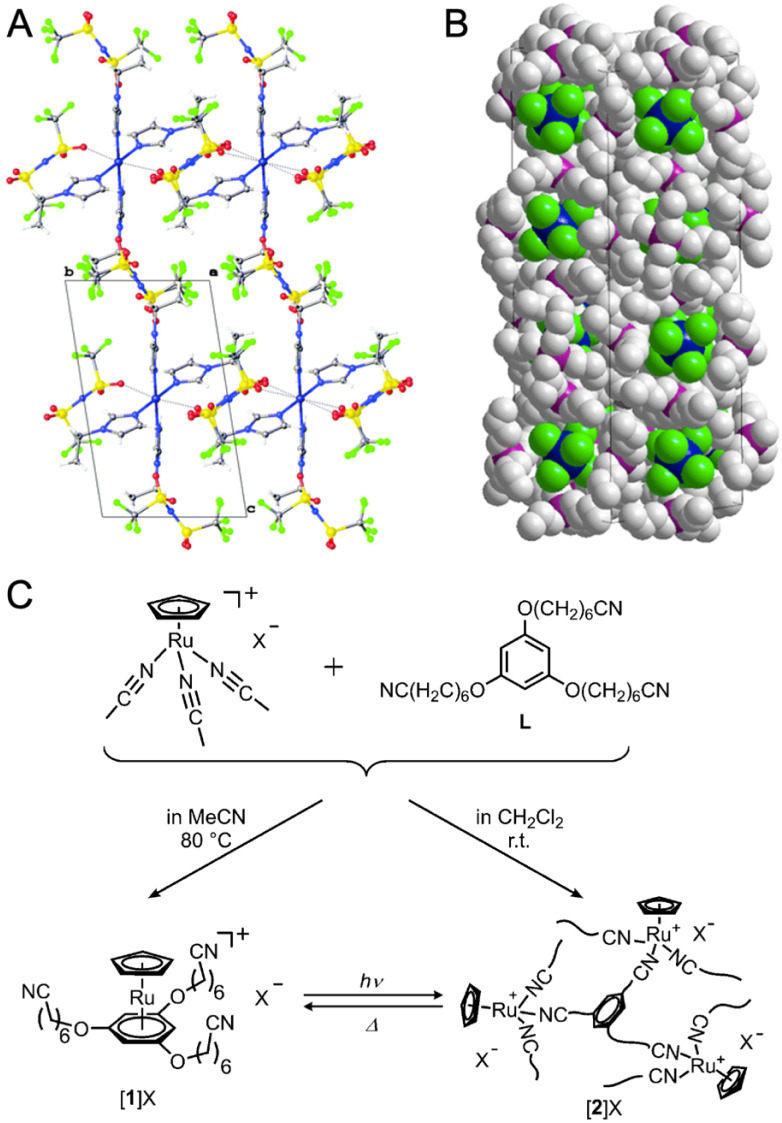
(A) View of the packing in the crystal structure of [Cu(*N*-ethylimidazole)_4_][Tf_2_N]_2_. (B) Packing in the large unit cell of [P_4444_]_3_[EuCl_6_]. (C) Reactions of [Ru(C_5_H_5_)(MeCN)_3_]X (X = FSA^−^, PF_6_^−^) and L. Conversion between [1]X and [2]X occurs for X = FSA^−^. (A) is adapted with permission from ref. 88. Copyright 2016 The Royal Society of Chemistry. (B) is adapted with permission from ref. 92. Copyright 2016 American Chemical Society. (C) is adapted with permission from ref. 93. Copyright 2016 The Royal Society of Chemistry.

The *T*_m_ of Co^2+^, Ni^2+^, and Cu^2+^ containing ionic liquids, [M(AlkIm)_*n*_][Tf_2_N]_2_ (AlkIm = *N*-alkylimidazole; Tf_2_N = bis(trifluoromethylsulfonyl)imide; *n* = 4 or 6), are influenced by both the length of the alkyl chain and the choice of cation on the *N*-alkylimidazole ligands (Fig. 10A).^88^ By adjusting the alkyl chain length on the *N*-alkylimidazole ligands in the [Cu(AlkIm)_4_][Tf_2_N]_2_ complexes, it becomes possible to modulate the *T*_m_ and alter their crystal structure due to the entropic contributions. This variability allows for the manipulation of the *T*_m_, ranging from 89 °C down to a liquid state below room temperature. For example, in the [Cu(AlkIm)_4_][Tf_2_N]_2_ series, the melting decreases from 89 °C for *N*-methylimidazole to 74 °C and 46 °C for *N*-ethylimidazole and *N*-butylimidazole to below room temperature for *N*-hexylimidazole. The choice of metal ion in the octahedral [M(MeIm)_6_][Tf_2_N]_2_ ionic liquids has a significant impact on their *T*_m_, where a lower *T*_m_ is observed when Cu^2+^ is utilized instead of Co^2+^ or Ni^2+^ due to the Jahn–Teller effect.

A series of alkyl-phosphonium ionic liquids with rare-earth elements, specifically [PR_4_]_3_[RECl_6_] (R = alkyl), behave differently at room temperature depending on the choice of the alkyl group.^92^ The alkyl-phosphonium cations include [P_666 14_]^+^, [P_4448_] ^+^, and [P_4444_] ^+^, while the rare-earth (RE) elements include La^3+^, Ce^3+^, Pr^3+^, Nd^3+^, Sm^3+^, Eu^3+^, Gd^3+^, Tm^3+^, Tb^3+^, Dy^3+^, Ho^3+^, Er^3+^, Yb^3+^, Lu^3+^, Y^3+^, and Sc^3+^. The [P_666 14_]_3_[RECl_6_] compounds containing rare-earth elements remain in a liquid state at room temperature with *T*_m_ between −58 and −40 °C, except for the La-containing compound, which solidifies at −1.6 °C. On the other hand, the [P_4444_]_3_[RECl_6_] compounds are solid and melt at temperatures between 43 and 103 °C. For the [P_4448_]^+^ cation, the heavier lanthanides have lower *T*_m_ between −6 and −48 °C, while the lighter lanthanides behave as supercooled liquids when cooled between 12 and 18 °C. These liquids are thermally stable up to 340–380 °C. The crystal structure of [P_4444_]_3_[EuCl_6_] contains [EuCl_6_]^3−^ anions surrounded by three crystallographically independent [P_4444_]^+^ cations. The packing arrangement in the large cell is shown in Fig. 10B, with space group *P*4_1_2_1_2_1_ and dimensions *a* = 16.5192(6) Å, *b* = 16.5192(6) Å, and *c* = 46.471(3) Å. X-ray absorption spectroscopy (XAS) of liquid samples of [P_666 14_]^+^ cations and [LnCl_6_]^3−^ anions (Ln = Nd^3+^, Eu^3+^, and Dy^3+^) confirmed that the lanthanide ions are hexa-coordinated by six chloride ligands in the liquid state. The EXAFS measurements revealed that the Ln⋯Cl distance decreases as the lanthanide ionic radius decreases: 2.70 Å (270 pm) for Nd^3+^ (ionic radius = 98.3 pm), 2.66 Å (266 pm) for Eu^3+^ (ionic radius = 94.7 pm), and 2.65 Å (265 pm) for Dy^3+^ (ionic radius = 91.2 pm). This decrease in distance is attributed to the stronger attraction between the lanthanide ion and the chloride ligands due to the increasing charge density.

A series of metal-containing ionic liquids, composed mainly of cationic organometallic sandwich complexes with nitrile-based anions, transform into CPs when exposed to light and revert to a liquid state through melting (Fig. 10C).^93–96^ Combining [Ru(C_5_H_5_)(MeCN)_3_]X (X = FSA^−^ or PF_6_^−^) with 1,3,5-C_6_H_3_(OC_6_H_12_CN)_3_ ligands provides colorless liquid or yellow amorphous CP, depending on the synthetic condition.^93^ Exposing the ionic liquid to UV light induces polymerization, while heating the solid at 90 °C for 30 min or 130 °C for 1 min reverses this process. Substituting the cation and anion with [Ru(C_5_H_5_)[C_6_H_3_(OC_6_H_12_CN)_3_]]^+^ and anionic covalent chain [CH_2_–CH(SO_2_N–SO_2_CF_3_)]_*n*_ results in a photoresponsive poly(ionic liquid). In this material, the ionic conductivity is reversibly controlled based on the mobility of cations, with polymerized states having lower mobility.^96^ [Ru(C_5_H_5_)(C_6_H_5_R)][B(CN)_4_] (R = butyl, ethyl, octyl) ionic liquids undergo polymerization triggered by UV irradiation.^94^ The process requires the elimination of the arene ligand, resulting in the formation of an amorphous CP Ru(C_5_H_5_)[B(CN)_4_] with microporosity.

### 2.4 Porous liquid

**Fig. 11:**
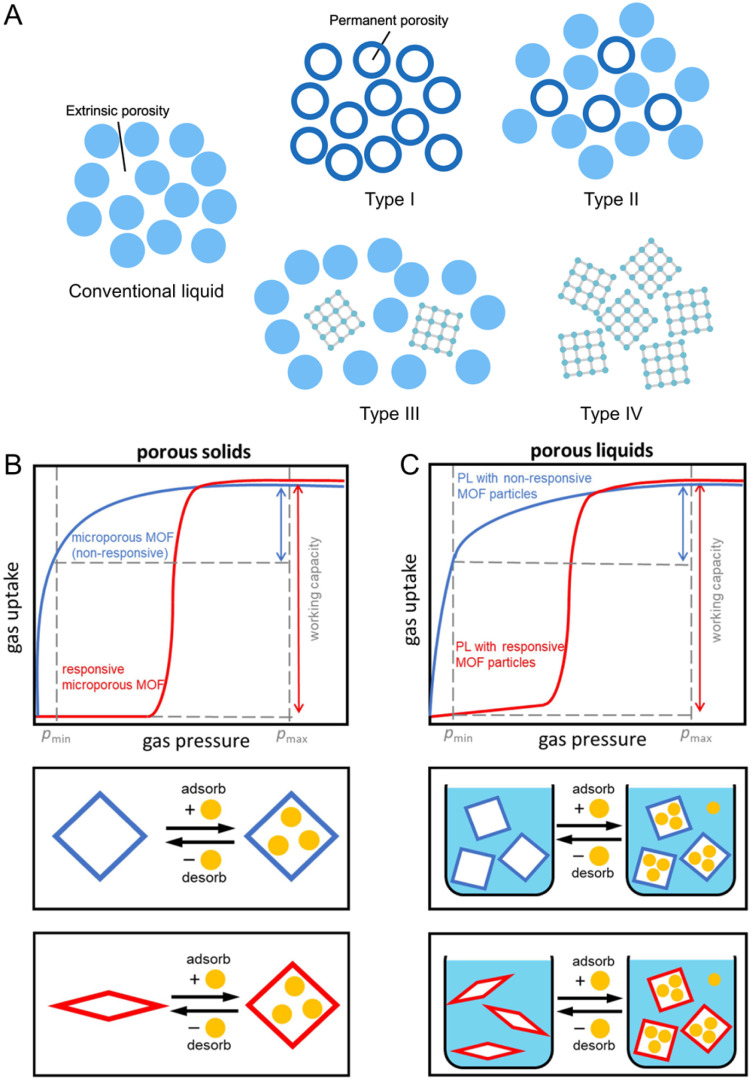
(A) Four types of porous liquids compared to conventional non-porous liquids. Type I: neat liquid hosts that cannot collapse or interpenetrate. Type II: empty hosts dissolved in sterically hindered solvents. Type III: framework materials dispersed in hindered solvents. Type IV: porosity retention in melt state of CPs/MOFs. (B) Sorption isotherms of non-responsive (blue) and responsive (red) porous solids. (C) Isothermal gas uptake of porous liquids with non-responsive CP/MOF particles (blue) and responsive CP/MOF particles (red). Maximum adsorption pressure (*p*_max_) and minimum desorption pressure (*p*_min_) for a pressure swing are indicated by dashed vertical grey lines. Adapted with permission from ref. 127. Copyright 2023 Springer Nature Limited under Creative Commons license CC BY 4.0. https://creativecommons.org/licenses/by/4.0/.

Generally, gases are stored in conventional liquids by dissolving and residing within intermolecular voids (Fig. 11A). The solubility of CO_2_ in water is only 0.04 M, while values of 0.27 and 6.97 M are achievable in acetonitrile and propylene carbonate, depending on the physical nature of the liquid hosts.^122,123^ Substituting saline water with fluorocarbons also increases O_2_ solubility by at least ten times and is promising for use as artificial blood.^124^ In addition to small transient cavities or extrinsic porosity, liquids with well-defined cavities will further improve gas solubility and, at the same time, selectivity. This concept of liquids with permanent porosity was proposed in 2007.^125^ Later on, the example of porous liquid was demonstrated in 2015.^126^ These microporous liquids possess intrinsic porosity, characterized by permanent, empty, and well-defined cavities within the molecules of the liquid or particles dispersed within it. These cavities provide potential guests with access to the interior of the liquid. Initially, porous liquids were classified into three distinct types (Fig. 11A).^125^ The discovery of meltable CPs/MOFs that maintain microporosity in their liquid phase introduced a new porous liquid category, Type IV.

Developing Type I CP/MOF-based porous liquids presents two primary challenges: lowering their *T*_m_ and preventing pore blockage by functional groups or guest molecules.^128^ Surface modification using liquefying agents such as polyethylene glycols and imidazoliums lowers the *T*_m_ of CPs/MOFs to below room temperature. Ion-exchanging of Cl^−^ in imidazolium-functionalized Deim-UiO-66 with a negatively charged poly(ethylene glycol)-tailed sulfonate (PEGS) canopy yields a stable Im-UiO-PL porous CP/MOF liquid. In contrast, the same process does not apply to a neutral UiO-66, highlighting the importance of cationic nature. The elongated carbon chains linked to the imidazolium groups functioned as protective coronas, ensuring the host cavities remained unblocked and accessible. The Im-UiO-PL exhibits slightly higher *T*_g_, *T*_c_, and *T*_m_ values of −51 °C, −6 °C, and 28 °C compared to PEGS, which has corresponding values of −53 °C, −14 °C, and 23 °C, respectively. MD simulations also supported the presence of permanent porosities ranging from 4 Å to 6 Å, which readily accommodate CO_2_ molecules. DFT analysis showed that PEGS has dimensions of 23.0 Å in length, 14.7 Å in width, and 20.2 Å in height, making it too large to enter Deim-UiO-66 cavities. As a result, Im-UiO-PL has 14 times greater CO_2_ adsorption capacity than pure PEGS.

Outer surface functionalization of crystalline ZIF-67 (Co(2-methylimidazolate)) with *N*-heterocyclic carbene ligands (NHCs) yields a Type III porous liquid.^129^ ZIF-67 particles with an average size of 264 ± 54 nm were synthesized and then surface grafted to the open metal site with 1,3-bis(2,4,6-trimethylphenyl)imidazole-2-ylidene (IMes) and 1,3-bis(2,4,6-diisopropylphenyl)imidazole-2-ylidene (IDip) NHCs. Dispersing IDip-modified ZIF-67 in cyclohexane, cyclooctane, and mesitylene yields a Type III porous liquid in which the overall uptake capability per gram of ZIF-67 is maintained. Stable dispersion also leads to the opportunity to co-process the ZIF-67 with the 6FDA-DAM polymer to create a mixed matrix membrane. A maximum filler loading of up to 47.5 wt% is achievable while preventing agglomeration.

Instead of using sterically bulky solvents, a stable dispersion of CPs/MOFs in water is achieved while maintaining a dry internal micropore.^130^ Several nanocrystalline zeolite and CPs/MOFs synthesized with hydrophobic pore surfaces excluded liquid water from their micropores at ambient temperature and pressure (entropically disfavored). In the case of zeolite, namely silicate-1, optimizing the synthetic condition of nanocrystalline alone yields a stable, translucent aqueous colloidal solution (38 wt%). This is explained by the nature of silicate-1, which has hydrophobic internal and hydrophilic external surfaces. The presence of dry micropores has led to an order of magnitude higher gas uptake: 26 ± 1 mmol O_2_ l^−1^ at 0.84 bar and 284 ± 2 mmol CO_2_ l^−1^ at 0.67 bar, as compared to 1.1 mmol O_2_ l^−1^ and 23 mmol CO_2_ l^−1^ for water under the same condition. Surface functionalization of Zn(mIm)_2_ (ZIF-8, mIm = 2-methylimidazolate) and Co(mIm)_2_ (ZIF-67), with surface ligands promotes dispersibility without blocking access to the micropore, leading to microporous water. Attaching bovine serum albumin (BSA) globular water-soluble proteins onto the ZIF-8 and ZIF-67 hydrophobic external surfaces could provide a complete dispersion with 80% ± 9% of the O_2_ theoretical capacity for ZIF-67. Aside from the non-covalent approach, reacting ZIF-8 with methoxypolyethylene epoxide (mPEG; *M*_n_ = 750 g mol^−1^ for PEG) results in an epoxy ring opening and mPEG functionalization. The measured O_2_ capacity of functionalized ZIF-8 (7.0 wt%) here is 96% ± 7% of the theoretical amount.

A dispersion of flexible CPs/MOFs, capable of structural changes under varying gas adsorption pressures, within a bulky solvent generates breathable porous liquids (Type III).^127^ While many CPs/MOFs exhibit a microporous structure with Langmuir-shaped gas sorption isotherms, the transition from a contracted, minimally porous phase to an expanded, highly porous phase in response to gas pressure results in sigmoidal adsorption profiles. These sigmoidal adsorption profiles persist in the porous liquid when breathable CPs/MOFs are dispersed in the bulky solvent (Fig. 11B). Responsive CPs/MOFs display significant gas uptake variations within a narrow pressure range, thus enhancing their working capacity. For instance, ZIF-7 and ZIF-9, known for their breathing behavior and featuring submicro- or nanoparticle sizes, were uniformly dispersed in silicone oil (1,3,3,5-tetramethyl-1,1,5,5-tetraphenyl-trisiloxane, Silicone 704, viscosity of 42 mPa s at 25 °C) to create breathable porous liquids upon 15 minutes of sonication. In particular, 5 wt% and 10 wt% ZIF-7 in Silicone 704 are denoted as PL7_5 and PL7_10, with the latter displaying particle sizes of 469 ± 74 nm as determined by DLS analysis and a viscosity of 107 mPa s at a shear rate of 10 s^−1^. ZIF-7 nanocrystals exhibit a CO_2_ adsorption capacity of approximately 43 cm^3^ (STP) g^−1^ at 1224 mbar and 25 °C, characterized by a sigmoidal sorption isotherm, while Silicone 704 displays a CO_2_ uptake of 1.2 cm^3^ (STP) g^−1^ under the same conditions. The CO_2_ sorption isotherms of PL7_10 and PL7_5 also display sigmoidal characteristics, with CO_2_ uptakes of 5.24 cm^3^ g^−1^ and 3.12 cm^3^ g^−1^, respectively.

**Fig. 12:**
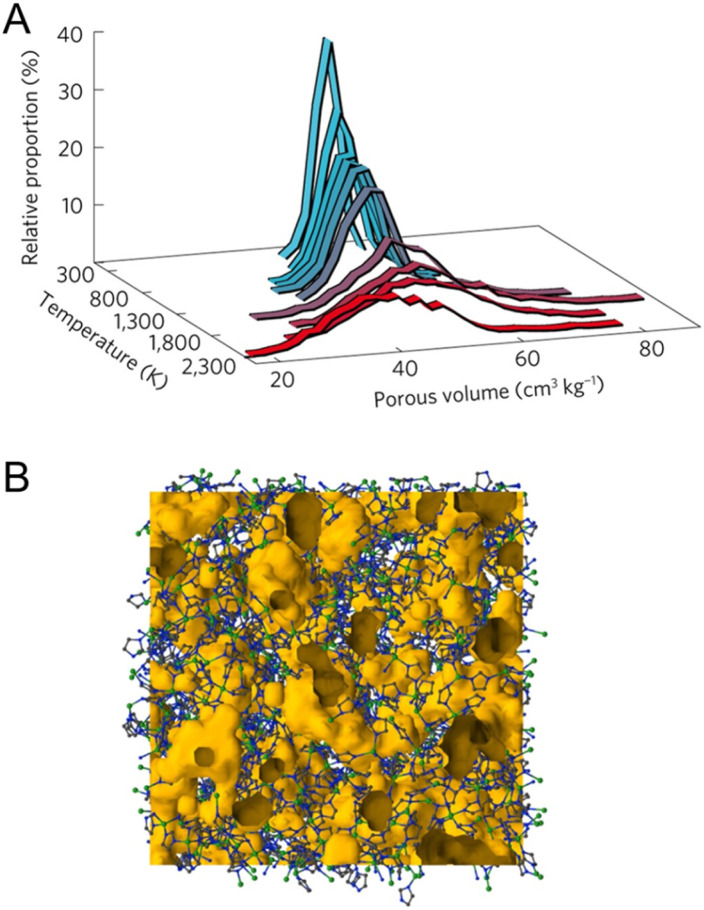
(A) Temperature evolution of the distribution of the total pore volume, determined for a standard probe of radius 1.2 Å. The average pore volume takes the following values: 52 cm^3^ kg^−1^ at 27 °C, 49 cm^3^ kg^−1^ at 1727 °C, and 41 cm^3^ kg^−1^ at 1977 °C. (B) Atomic configuration of the ZIF melt, gained from reverse Monte Carlo modeling of the total scattering data collected at 583 °C. Free volume is represented in orange, Zn atoms in green, N in blue, and C in grey. Adapted with permission from ref. 29. Copyright 2017 Springer Nature Limited.

Without any bulky solvent, porosity preservation in network-forming ZIF-4 above the *T*_m_ leads to a new category of porous liquid (Type IV).^29^ Upon melting, the translational diffusion of Zn^2+^ and imidazolate ions is calculated to be 7.7 × 10^−10^ m^2^ s^−1^ and 6.5 × 10^−10^ m^2^ s^−1^, respectively, at temperature of 1227 °C (FPMD). Note that the higher temperature in FPMD is not physically relevant for the experimental system, as mentioned in an earlier section. The viscosity of 19 mPa s at 1227 °C is estimated from these diffusion coefficients. Extrapolating by the Arrhenius law, the viscosity at the experimental *T*_m_ of 567 °C is equivalent to 8000 mPa s. A slight variation in porosity development is observed in the solid phase at temperatures below 927 °C. When the system transitions to the liquid state at higher temperatures, the overall porosity remains relatively consistent, with a slight shift towards lower average pore volumes. 95% of the void space in ZIF-4 liquid at 1227 °C is accessible porosity, while crystalline ZIF-4 voids are 74% accessible at 27 °C, suggesting a hybrid porous liquid even at high temperatures (Fig. 12). The porosity of the ZIF-4 porous liquid is also larger than that of similar imidazolium ionic liquids, where the void space size distribution is typically negligible above 1 Å in radius. RMC modeling on X-ray total scattering data was performed, and the internal surface of the glass was calculated using a standard probe diameter of 2.4 Å. The results suggest that the internal surface was enhanced to 16.2% in the liquid phase at 583 °C from 4.8% in glass at ambient temperature, referring to the intrinsically porous liquid of melt ZIF-4.

In addition to meltable CP/MOF systems, retention of permanent porosity is observed in the liquid state of discrete coordination cages and potentially in metal–organic polyhedra (MOPs).^84–86^ Attaching long poly(ethylene glycol)-imidazolium chains into the periphery of the parent Zn_4_L_4_ tetrahedron (methylated ligand) results in a room-temperature ionic liquid with permanent porosity.^84^ A stable liquid is maintained between −44 and 300 °C and has an average void diameter of 6.29 ± 0.08 Å at 298 K (*ortho*-positronium lifetime of 2.34 ± 0.05 ns), which accommodates gaseous chlorofluorocarbon and non-gaseous alcohol molecules. Surface grafting of amine-terminated poly(ethylene glycol) onto carboxylic acid-functionalized Rh(ii)-based MOPs *via* covalent amide formation enables melting behavior (*T*_m_ = 47 °C).^85^ Without the intrusion of the surface polymer chains, the porosity of the MOPs is maintained in amorphous form. This allows the use of meltable MOP as a matrix for creating mixed-matrix composite films with porous MOFs. Another example of melting behavior is enabled by grafting MOP with tethered polymers onto open metal sites (axial positions) through a coordination bond (*T*_m_ = 47 °C).^86^ The polymer design inhibits the interpenetration of polymers into the MOP's internal cavity. One of the terminals contains a Lewis-basic coordination site to bind with the MOP. The other side has a bulky functional group that is larger than that of the pore opening. Cooling the liquid state faster than 30 °C min^−1^ results in a glass transition at −56 °C. Subsequent heating induces crystallization (*T*_c_ = −29 °C). Moreover, the thermal behaviors are controllable by tuning the polymer length and the polarity on the MOP surface (functional group substituent). With *T*_m_ between 17 and 25 °C, they behave as porous liquids at room temperature with maintained porosity and gas-separating capability even in liquid forms.

## 3. Crystal-liquid transition

Reversible phase change is perhaps a more ubiquitous phenomenon than the thermodynamic well, which is the glassy state. Many of us are familiar with the melting and recrystallization of simple organic molecules. In the case of melting CPs/MOFs, if the appropriate conditions are met, cooling the melt will result in recrystallization of the pristine crystal structure rather than the trapping of the glassy state. From an application point of view, this provides CPs/MOFs with the capability to process and shape them into complex structures in the liquid phase, and then functional crystalline monoliths are later obtained by cooling below the crystallization temperature. Therefore, it is crucial to understand what influences the reversibility of the crystal-liquid phase change in CPs/MOFs.

**Fig. 13:**
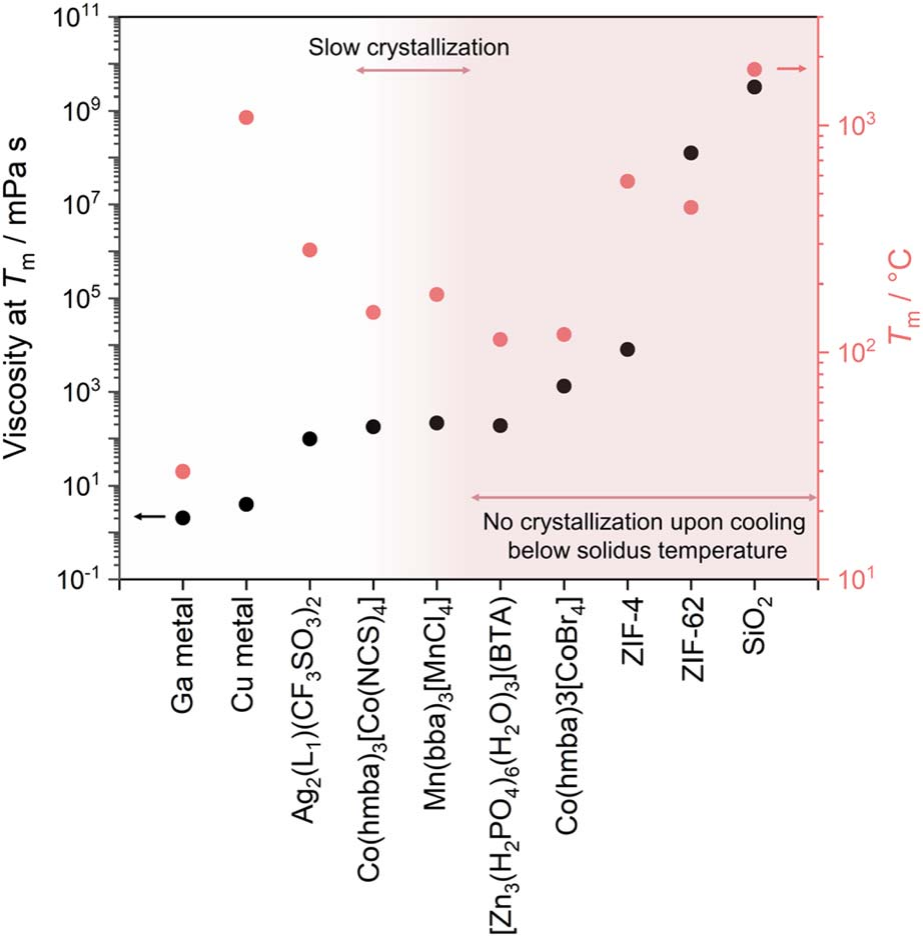
Comparison of the viscosity at *T*_m_ and behavior upon cooling of different meltable CPs/MOFs.

A series of metal-bis(acetamide) frameworks has been investigated to gain insight into their phase transitions.^45^ These ligands are advantageous for producing melting CPs/MOFs as they feature weaker, more labile coordination bonds (*i.e.*, the O atom of the acetamide moieties) and longer aliphatic bridging sections that offer greater ligand flexibility. These properties work together to decrease Δ*H* and increase Δ*S*, respectively, resulting in lowered *T*_m_. The kinetics of recrystallization are affected most noticeably by the *T*_m_ and viscosities of the materials. Mn(bba)_3_[MnCl_4_], Mn(bba)_3_[ZnCl_4_], Mg(bba)_3_[CoCl_4_], and Mg(bba)_3_[ZnCl_4_] all exhibit fast recrystallization upon cooling of the melt, while at the same time, they have some of the highest *T*_m_ of the compounds in the study. This gives the compounds ample energy to overcome Δ*H*_fus_. The compound in this series with lower *T*_m_ (and relatively higher entropies) tended to show slow recrystallization kinetics, where fast cooling resulted in the glassy state and isothermal hold was needed to recrystallize the compound completely. Co(hmba)_3_[CoBr_4_] has the lowest *T*_m_ and high viscosity in the melt (1313 mPa s at 120 °C, Fig. 13), which, coupled with retained anions and cationic networks in the melt, hinders its ability to recrystallize completely. Varying M′ gave a more marked difference in *T*_m_ than varying the corresponding [M′′X_4_]^2−^, due to M′ being the main framework metal ions. Changing the linker from the shorter bba to the longer hmba expectedly increased the Δ*S*_fus_, as the longer linker is more flexible, while also decreasing the *T*_m_.

Ag_2_(L1)(CF_3_SO_3_)_2_ (L1 = 4,4′-biphenyldicarbonitrile), a Ag^+^-based CP, shows *T*_m_ of 282 °C and crystallizes rapidly upon cooling to below 242 °C.^42^ The Δ*H*_fus_ and Δ*S*_fus_ of Ag_2_(L1)(CF_3_SO_3_)_2_ were 11.4 kJ mol^−1^ and 20.5 J mol^−1^ K^−1^, respectively. Rheological measurements revealed that Ag_2_(L1)(CF_3_SO_3_)_2_ has a viscosity of 98 mPa s at 282 °C (*T*_m_), which is remarkably low compared to some other melting CPs/MOFs such as ZIF-62 (10^8.1^ mPa s) or the aforementioned Co(hmba)_3_[CoBr_4_] (1313 mPa s). The high *T*_m_, giving sufficient energy for nucleation, coupled with the low melt viscosity, are the major contributors to the rapid recrystallization of [Ag_2_(L1)(CF_3_SO_3_)_2_]. Cycling through melting and crystallization cycles results in morphological changes, resulting in the evolution of melting endotherms with continuous *T*_m_ lowering. Analyzing crystallization kinetics using the Avrami equation yields Avrami exponents falling within the range of 2.27 to 2.32. The value suggests a 1D rod-like crystal growth mechanism arising from the sporadic nuclei, aligning with the observations in DSC, VT-PXRD, Raman spectroscopy, real-time hot-stage microscopy, and polarized optical microscopy.

**Fig. 14:**
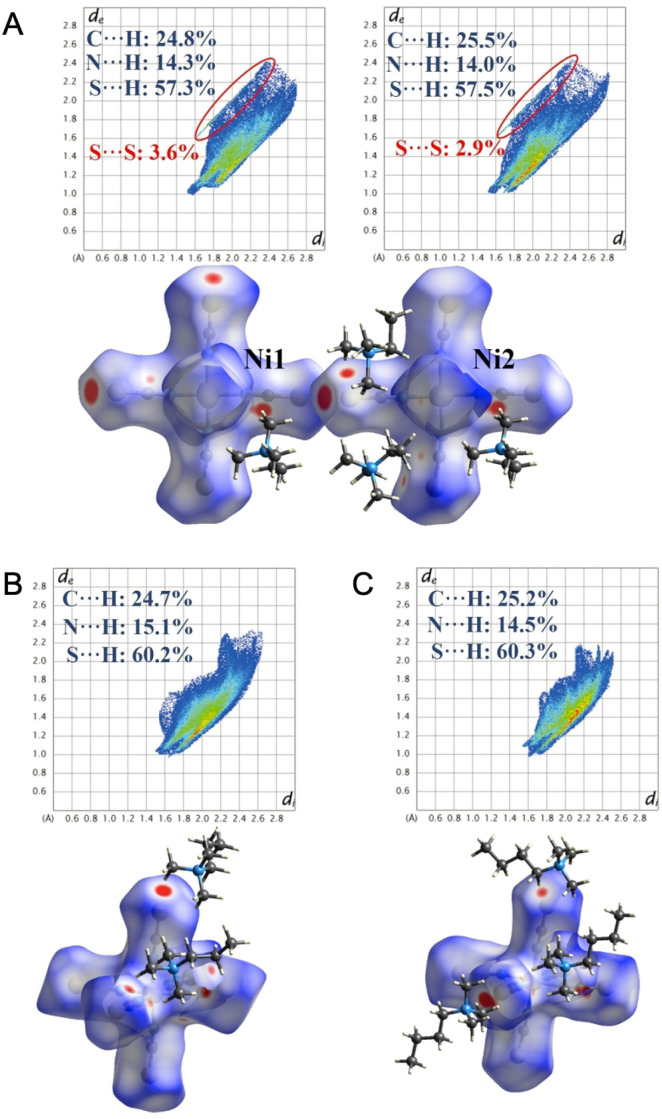
The Hirshfeld surfaces mapped with *d*_norm_ and fingerprint plots for crystallographically independent [Ni(NCS)_6_]^4−^ anions in (Me_3_NR)_4_[Ni(NCS)_6_], R = (A) ethyl, (B) propyl, and (c) butyl. Adapted with permission from ref. 131. Copyright 2023 John Wiley & Sons, Inc.

The phase-transition behavior is studied in a series of hybrid organic–inorganic crystals of the type (Me_3_NR)_4_[Ni(NCS)_6_], where R = ethyl, propyl, or butyl.^131^ The three compounds show multiple solid–solid phase transitions in a wide temperature range from −113 to 140 °C, while the propyl and butyl substituted compounds also exhibit melting and recrystallization behavior. The Hirshfeld surfaces of the various compounds and their respective crystalline phases were examined to gain a deeper understanding of the underlying intermolecular interactions responsible for the phase changes (Fig. 14). In the crystal containing ethyl-substituted amines, the [Ni(NCS)_6_]^4−^ anions interact with adjacent anions through S⋯S short contacts, adding to the overall attractive forces holding the crystal together. The small relative size of Me_3_NEt^+^ also improves attractive Coulomb interactions between the cations and anions. The two melting crystals containing propyl or butyl groups did not have the same S⋯S short contacts as in the ethyl-substituted compounds. As such, only Coulomb interactions needed to be overcome in these compounds, which resulted in melting.

**Fig. 15:**
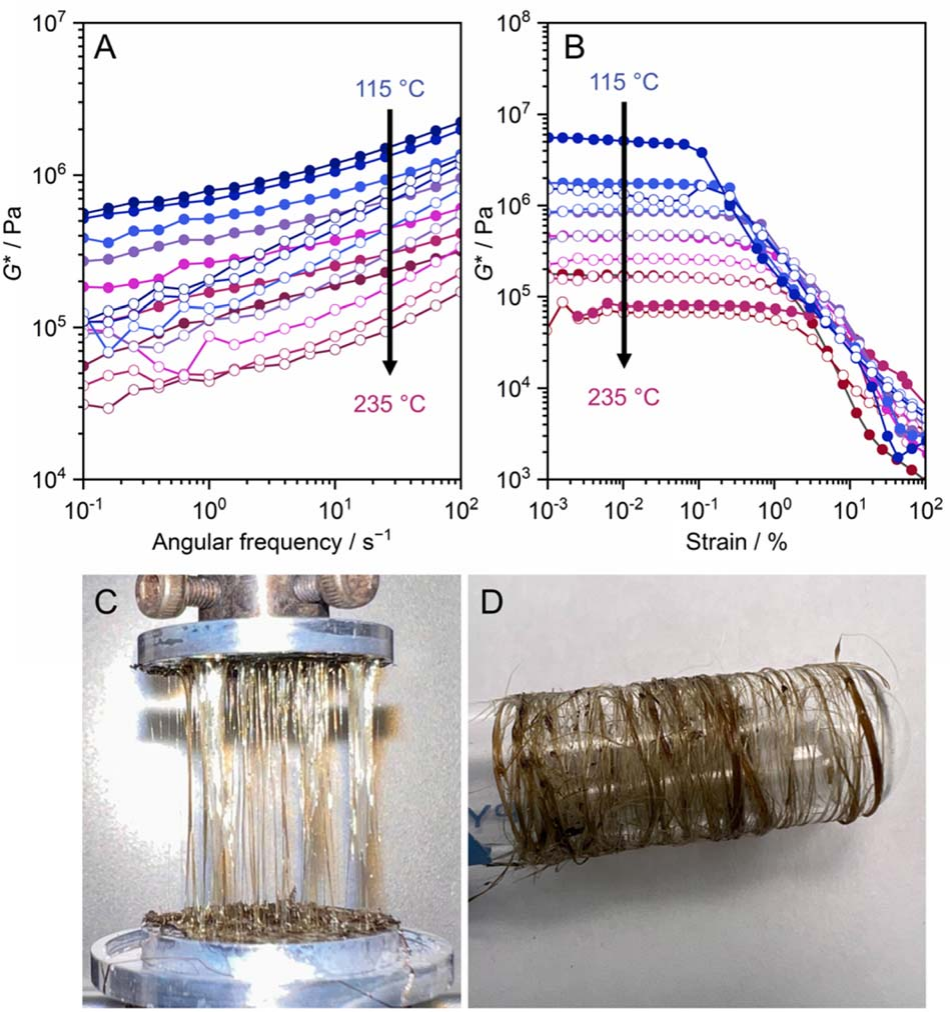
(A) Frequency sweep and (B) strain sweep viscoelastic measurements of 1D Cu(2-isopropylimidazolate) from 235 to 115 °C under N_2_ flow. The filled and opened circles represent *G*′ and *G*′′, respectively. (C) Fibers of Cu(2-isopropylimidazolate) prepared by hot-pressing and collected by spinning around the glass tube. Adapted with permission from ref. 30. Copyright 2021 The Royal Society of Chemistry. https://creativecommons.org/licenses/by/3.0/.

Cu(2-isopropylimidazolate) forms a melt state above 143 °C and recrystallizes below 112 °C, showing good consistency in the reversibility of this transition.^30^ The CP does not become glass, even upon rapid cooling to −196 °C from the melt state. A major contributing factor to this may be the molecular structure of liquid Cu(2-isopropylimidazolate). PDF analysis above the *T*_m_ suggests retention of Cu^+^-isopropylimidazolate-Cu^+^ bridging motifs in the liquid phase. This is also supported by rheological studies that show a large *G*′ (*ca.* 10^6^ Pa) above the *T*_m_, indicating a high viscosity and preservation of 1D structure in the liquid state (Fig. 15). Although high viscosity has been shown to limit recrystallization in some CPs, the high viscosity in the case of Cu(2-isopropylimidazolate) is due to its retention of molecular structure, so cooling of the melt results in less thermal motion of the 1D chains that again return into a crystalline solid.

## 4. Functions and applications

### 4.1 Ionic conductivity

Transitioning between phases in CP/MOF yields distinct advantages in terms of ionic conductivity, encompassing enhancements in molecular dynamics at the microlevel and facilitating processability at macroscales. An early example of enhanced conductivity after phase transition was demonstrated in a non-melt 2D layered Cd(H_2_PO_4_)_2_(1,2,4-triazole)_2_.^68^ The crystal-to-glass transition *via* mechanical vitrification leads to enhanced anhydrous proton conductivity over two orders of magnitude, reaching 1 × 10^−4^ S cm^−1^ at 125 °C. The behavior arises as the acidity and isotropy of H_2_PO_4_^−^ increase, leading to higher overall proton mobility. The event is reversible. As heating continues above the crystallization temperature (142 °C), the conductivity value reverts as recrystallization occurs.

**Fig. 16:**
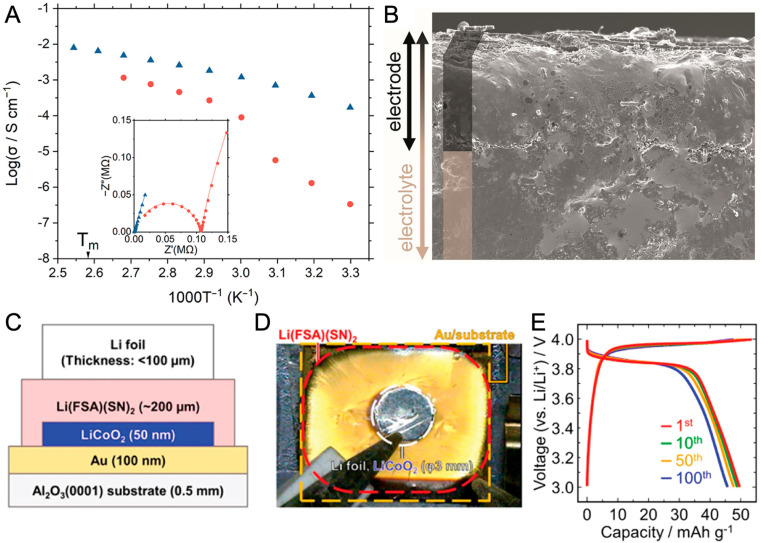
(A) Arrhenius plots of the anhydrous H^+^ conductivity of a [Zn_3_(H_2_PO_4_)_6_(H_2_O)_3_](BTA) degas crystal (●) and MQG (▲) under an Ar atmosphere. (B) Cross-sectional SEM images (150× magnification) of the electrode–solid-state electrolyte interface. Adapted with permission from ref. 32. Copyright 2021 The Royal Society of Chemistry. https://creativecommons.org/licenses/by/3.0/. (C) Cell configuration and (D) top-view photograph of Li-battery (Li|Li[N(SO_2_F)_2_](NCCH_2_CH_2_CN)_2_|LiCoO_2_|Au). (E) Charge–discharge profiles at a current density of 1 μA cm^−2^. Adapted with permission from ref. 37. Copyright 2020 American Chemical Society.

Another advantage of the reversible solid–liquid transition is its shaping capability, which allows for versatile and adaptable formability. The 1D meltable and proton-conductive [Zn_3_(H_2_PO_4_)_6_(H_2_O)_3_](1,2,3-benzotriazole) is an example of the case.^32^ The compound exhibits melting behavior with a *T*_m_ of 114 °C and is quenched to form glass upon subsequent cooling. The glass behaves like a rigid solid below 90 °C, where its viscosity lies above the Littleton softening point (10^6.6^ Pa s). Above 90 °C, the viscosity drops to 42.8 Pa s (120 °C), which is below the working point of the glass. This allows the compound to be shaped above 90 °C and used as a solid below that temperature. Note that the working point for soda-lime-silica glass is above 1100 °C and is suitable for industrial processing.^111^ Increases in overall proton conductivity were observed throughout the measurement range (Fig. 16A). An approximately six-fold higher conductivity is observed at 60 °C, while the highest value of 8 × 10^−3^ S cm^−1^ is achieved at 120 °C. A prototype solid-state proton battery using glass as a solid electrolyte shows an indistinguishable electrode–electrolyte interface and the absence of grain-boundaries, confirmed by cross-sectional SEM images (Fig. 16B). The solid-state proton battery shows a discharge capacity of 55.4 mA h g^−1^ at 25 °C and works up to 110 °C. In another work, the absence of grain boundaries also contributed to the enhanced ion transport of Li-ion electrolyte guests when comparing the glass with crystalline ZIF-4 hosts.^132^

Reversible crystal-liquid transition without forming a glass also aids device fabrication. 3D tetrahedrally coordinated Li[N(SO_2_F)_2_](NCCH_2_CH_2_CN)_2_ conduct Li-ion with conductivity values of 1 × 10^−4^ S cm^−1^ at 30 °C and 1 × 10^−5^ S cm^−1^ at −20 °C with a transport number of 0.95.^37^ All solid-state Li-ion batteries were fabricated upon melting and used after crystallization (Fig. 16C–E). The fractures of the solid-electrolyte layer formed during operation are repairable by repeating the melt-crystallize cycle.

**Fig. 17:**
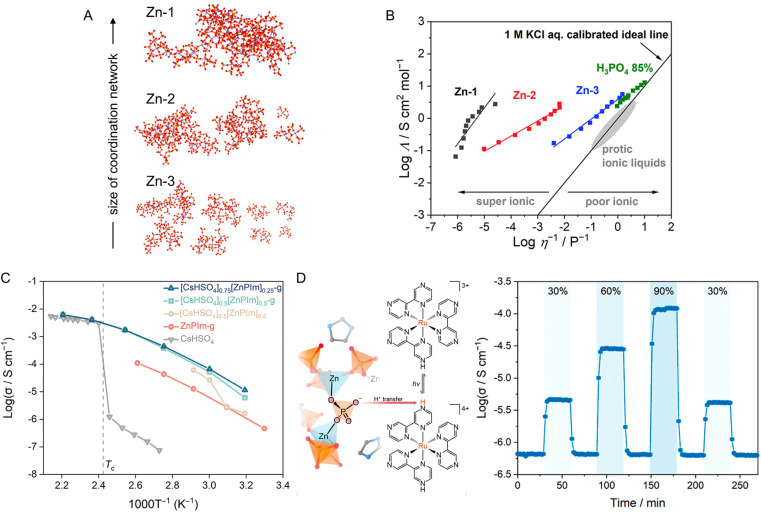
(A) Simulated coordination network structures (RMC) of dema_*x*_[Zn_*y*_(HnPO_4_)_3_]. The relative position and size of each structure are arranged for clarity. The Zn (blue), P (orange), and O (red) were shown as ball and stick models. H atoms were omitted for clarity. (B) Walden plots and the comparison with H_3_PO_4_ (85%) and protic ionic liquids. Adapted with permission from ref. 74. Copyright 2022 American Chemical Society. (C) Variable temperature anhydrous H^+^ conductivity of [CsHSO_4_]_0.75_[ZnPIm]_0.25_-g (blue), [CsHSO_4_]_0.5_[ZnPIm]_0.5_-g (green), [CsHSO_4_]_0.5_[ZnPIm]_0.5_ (physically blended, beige), ZnPIm-g (red), and CsHSO_4_ (gray). Reprinted with permission from ref. 107. Copyright 2023 American Chemical Society. (D) Schematic illustrating photoinduced protonation of monoprotonated Rubpz, generating H^+^-deficient sites in Rubpz-ZnPIm-g, and photoexcited H^+^ conductivity of Rubpz–ZnPIm-g at 30 °C under anhydrous condition. Light irradiation is demonstrated by blue highlighting, where the percentage indicates the light intensity controlled by the equipped continuous variable neutral density (ND) filter. Adapted with permission from ref. 133. Copyright 2023 American Chemical Society.

Proton conductivity and viscosity are modulated through coordination network connectivity, as demonstrated in a series of dema_*x*_[Zn_*y*_(H_*n*_PO_4_)_3_] (Fig. 17A and B).^73,74^ They were directly synthesized from (dema)(H_2_PO_4_) protic ionic liquid (dema = diethylmethyl ammonium), where the Zn^2+^ contents control the size of the coordination network. At the highest Zn^2+^ concentration, the conductivity of (dema)_0.35_[Zn(H_2.22_PO_4_)_3_] reaches 1.3 × 10^−2^ S cm^−1^ at 120 °C under anhydrous conditions, with a proton transport number of 0.94. This is because the coordination network helps restrict the movement of counter anions. As the connectivity decreases, the viscosity decreases from 10^6^ to 10^1^ Pa s. While their proton conductivities slightly increase in a narrow range up to 2.1 × 10^−2^ S cm^−1^ at 120 °C in (dema)_0.45_[Zn_0.75_(H_2.35_PO_4_)_3_]. A higher proton conductivity does not always translate to higher power delivery in H_2_/O_2_ fuel cells, as the fuel crossover cannot be efficiently mitigated due to insufficient viscosity.

The wide composition range and reversible phase transition of ZnPIm are used as a suitable host matrix to maintain the high conductivity of the guest molecules.^107^ CsHSO_4_ is a superprotonic solid acid known for its fast proton conduction, but only above the transition temperature (*T*_c_) of 141 °C. By forming a binary CsHSO_4_–ZnPIm glass system (discussed in Section 2.2), their anhydrous conductivities below *T*_c_ are over three orders of magnitude higher than the CsHSO_4_ without compromising the conductivity above *T*_c_. At 180 °C, the conductivity reaches 6.3 × 10^−2^ S cm^−1^(Fig. 17C). The preservation of conductivity is attributed to the oxyanion exchange between HSO_4_^−^ and bridging phosphate. The event induces chain scission, thus increasing the overall molecular dynamics. Forming a binary glass system also introduces processing capability to the CsHSO_4_, as a micrometer-scale thin film was prepared with a transmittance over 85% between 380 and 800 nm.

The presence of a liquid phase plays a crucial role in facilitating the homogeneous distribution of guest molecules within the CPs/MOFs matrix. By adding trifluoromethanesulfonic acid (15 mol%) to ZnPIm upon melting, the proton conductivity of the crystalized sample increases to 2.0 × 10^−7^ and 2.7 × 10^−4^ S cm^−1^ from 3.2 × 10^−9^ and 2.1 × 10^−5^ S cm^−1^ for the pristine sample at 30 and 110 °C, respectively.^40^ Doping a photoresponsive molecule, such as trisodium 8-hydroxy-1,3,6-pyrenetrisulfonate (pyranine), enables overall conductivity control upon 365 nm light exposure. The system reaches equilibrium within *ca.* 50 min, where the overall resistance decreases by 1.7 times. Pyranine releases protons upon irradiation, increasing the number of charge carriers in the system and thus the conductivity. A reverse event occurs when the irradiation ends and overall conductivity decreases.

An alternative approach to achieving control over proton conductivity under anhydrous conditions is by generating proton-deficient sites within the host matrix (Fig. 17D). Photoexcitation of the tris(bipyrazine)-ruthenium complex (Rubpz, 0.1 mol%) leads to improved responsiveness and intensifies the conductivity change between on and off states.^133–135^ The system reaches the highest on/off ratio of over 180 times within 5 min, and the conductivity was controlled explicitly by continuously controlling the light intensity and ambient temperature. The metal-to-ligand charge transfer (MLCT) excitation of Rubpz initiates the p*K*_a_ changes, where the proton is transferred from the ZnPIm glass domain to Rubpz, thus generating proton-deficient sites. The behavior leads to a lower energy barrier (*E*_a_), from 0.76 to 0.30 eV, required to initiate proton migration without disturbing the local structure of the glass.

### 4.2 Gas absorption and permeability

Permanent porosity in some melt-quenched ZIF glasses has been reported.^136^ ZIF-76 glass structures, [Zn(Im)_1.62_(5-ClbIm)_0.38_] with 5-ClbIm representing 5-chlorobenzimidazolate (C_7_H_4_N_2_Cl^−^), maintain the porosity revealed by the positron annihilation lifetime spectroscopy (PALS) technique. ZIF-76 glass features a single 5 Å pore that adsorbs over 4 wt% of CO_2_ at 0 °C, but desorption requires prolonged equilibration, as seen in the hysteresis isotherm. This suggests limited guest molecule diffusion in the intricate pore network due to the partial collapse of distinct 5.7 Å and 15.7 Å cavities post-quenching. This aligns with low N_2_ and H_2_ adsorption at −196 °C. By adjusting linkers, matching pores and molecule sizes for gas separation were achieved. A methyl group in ZIF-76-mbIm glass anchors its structure through non-covalent interactions with nearby ligands, maintaining an interconnected network of pores and channels within the glass, leading to permanent porosity. ZIF-76-mbIm glass with two pore diameters of 4.8 Å and 7.2 Å displays reversible CO_2_ (0.12 cm^3^ g^−1^ at 0 °C, 1 bar) and CH_4_ adsorption at 20 °C with minimal hysteresis, indicating low diffusion limitations. Yet, N_2_ diffusion is hindered, and H_2_ adsorption shows hysteresis. Isosteric heats of adsorption suggest that vitrification creates sites in the pore network with high CO_2_ affinity. The network of channels in ZIF-76-mbIm glass has remained stable for three months. These characteristics are beneficial for gas absorption and permeability applications.

**Fig. 18:**
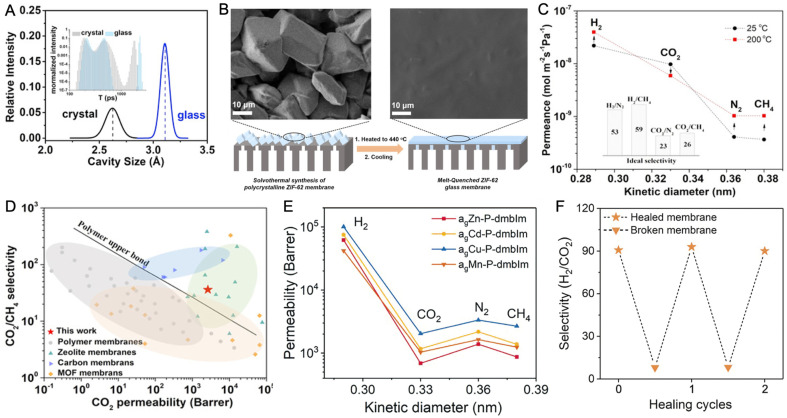
(A) Pore size distribution derived from PALS of ZIF-62 crystal and glass. (B) Schematic and top-view SEM images of polycrystalline ZIF-62 membrane and ZIF-62 glass membrane. (C) Single gas permeability as a function of the gas kinetic diameter of the ZIF-62 glass membranes at 25 °C and separation performance comparison with reported membranes. (D) CO_2_/CH_4_ separation performance comparison with reported membranes. Adapted with permission from ref. 137. Copyright 2020 John Wiley & Sons, Inc. (E) Single gas permeability as a function of the gas kinetic diameter from the a_g_M–P-dmbIm glass membranes at 20 °C and 1 bar. M represents Zn^2+^, Cd^2+^, Cu^2+^, and Mn^2+^; P is phosphate; and dmbIm is 5,6-dimethylbenzimidazole. (F) The H_2_/CO_2_ selectivity of a_g_Zn–P-dmbIm during the cyclic healing process. Adapted with permission from ref. 139. Copyright 2021 John Wiley & Sons, Inc.

Melt-quenching of the solvothermal synthesized ZIF-62 membrane on alumina support results in a monolithic membrane without a grain boundary that minimizes undesired interparticle diffusion, a feature particularly advantageous for gas separation.^137^ Post-quenching, PALS shows that the microporosity of ZIF-62 glass increases its radius to 3.16 Å compared to 2.66 Å found in the crystalline counterpart (Fig. 18A). At 20 °C and 1 bar, CO_2_, CH_4_, and N_2_ gas uptake decreases from 18.5, 10, and 2.4 cm^3^ g^−1^ in the crystalline state to 11, 2.6, and 0.7 cm^3^ g^−1^ in the glass state. The interaction between ZIF-62 and CO_2_ strengthens after converting to the glassy state, with an isosteric heat of adsorption (*Q*^0^_st_) of 29 kJ mol^−1^ compared to the original state of 26 kJ mol^−1^. A composite membrane of CP/MOF glass was created by melting a polycrystalline CP/MOF membrane on a porous ceramic alumina support at 440 °C for 15 minutes, enabling the molten substance to infiltrate the nanopores of the support through capillary extrusion. The glass membrane on the substrate is achieved after rapid cooling, where the gaps, pinholes, and grain boundaries are absent in the glassy ZIF-62 membrane (Fig. 18B). The ideal selectivity, or the ratios of the permeability of faster-moving gas to slower-moving gas, in the glass membrane for H_2_/N_2_, H_2_/CH_4_, CO_2_/N_2_, and CO_2_/CH_4_ pairs at 25 °C reaches values of 53, 59, 23, and 26, respectively (Fig. 18C). These values were far above the Knudsen selectivities (3.7, 2.8, 0.8, and 0.6), determined by the inverse square root of the molecular masses. In the case of single gas permeation, the activation energy of permeation increases with gas molecule sizes, which are 4.0, 6.1, and 6.8 kJ mol^−1^ for H_2_, CH_4_, and N_2_, respectively. In contrast, the activation energy of CO_2_ permeation is −2.6 kJ mol^−1^ as the CO_2_ permeance decreased with increasing temperature due to the high heat of adsorption for CO_2_ in ZIF-62 glass. For the binary gas mixture, including H_2_/CH_4_, CO_2_/N_2_, and CO_2_/CH_4_, the ZIF-62 glass membrane shows a promising gas separation ability. The selectivity of 50.7 is achieved for an equimolar mixture of H_2_/CH_4_ at 25 °C and 1 bar. Although the flexibility of CPs/MOFs compromises molecular sieving, the H_2_/CH_4_ separation efficiency of glass CP/MOF membranes exceeds that of the majority of reported polycrystalline CP/MOF membranes (Fig. 18D). The exceptional performance of the ZIF-62 glass membrane exceeded Robeson's upper boundary,^138^ which represents the tread-off relation between permeability and selectivity. While zeolite and carbon membranes exhibit strong H_2_/CH_4_ separation, they encounter issues such as physical aging and low reproducibility with grain boundary defects. The glass membranes offer comparable performance without these drawbacks. For CO_2_/N_2_ and CO_2_/CH_4_ equimolar gas mixtures at 25 °C and 1 bar, the glass membrane demonstrates CO_2_ selectivities of 34.5 and 36.6, respectively, surpassing ideal selectivity due to its greater CO_2_ adsorption than N_2_ and CH_4_. The CO_2_ permeabilities of the membranes for CO_2_/N_2_ and CO_2_/CH_4_ are 2602 and 2638 Barrer. These values also surpass Robeson's upper bound. The glass membrane exhibits resilience to water, although the gas permeance diminishes upon exposure to water vapor, leading to complete filling of the micropores within 24 hours. The restoration of empty micropores was achieved by subjecting the membrane to 180 °C for 2 hours, utilizing dry feeding gas and He sweep gas. Apart from CP/MOF glasses, the promising feasibility of upscaling the production of glass membranes also relies on well-suited supports.

While high vitrification temperatures and viscosity could limit the scalability of ZIF glasses, a series of low-viscosity meltable CPs (M–P-dmbIm) with *T*_m_ around 162–176 °C have been developed as free-standing glass membranes with a thickness of *ca.* 95 μm *via* the hot-casting method.^139^ Note that M represents Zn^2+^, Cd^2+^, Cu^2+^, and Mn^2+^; P is phosphate; and dmbIm is 5,6-dimethylbenzimidazole. The viscosity values of Zn–P-dmbIm are 45.0 and 61.2 Pa s at *T*_m_ (176 °C) and *T*_g_ (112 °C), respectively. This is much lower than ZIF-62, where much higher viscosity values of 10^5^ and 10^12^ Pa s are observed at *T*_m_ and *T*_g_. The low *T*_m_, *T*_g_, and low viscosity are a result of the formation of the framework through non-covalent interactions. The steric hindrance caused by the 5,6-dmbIm ligand also contributes to an exceptionally high glass forming ability (GFA) ranging from 0.85 to 0.94 in the M–P-dmbIm. This hinders the movement of atoms or ions, thus inhibiting the creation of orderly crystal formations after glass membrane fabrication. The PALS reveals the pore size diameter of 2.93 Å and 4.87 Å of Zn–P-dmbIm glass membrane decreased from 3.4 and 5.5 Å of its crystalline state, which is slightly larger than the H_2_ kinetic diameter (2.9 Å), while inhibiting larger gas penetration. The H_2_ permeabilities at 1 bar pressure of Zn-, Cd-, Cu-, and Mn–P-dmbIm are calculated to be 62 000, 75 000, 99 000, and 42 000 barrer, which are higher among other pure gases, including CO_2_, N_2_, and CH_4_ (Fig. 18E). Similar to ZIF-62 glass, the M–P-dmbIm glass membranes also demonstrate ideal selectivity exceeding Knudsen selectivity within binary gas mixtures. The selectivity of Zn–P-dmbIm glass membrane for H_2_/CO_2_, H_2_/N_2_, and H_2_/CH_4_ is 92.7, 49.6, and 75.0, respectively, with H_2_ permeability of 6.47 × 10^4^ barrer. Their separation ability also surpasses the Robeson upper bound, pointing to their potential applicability for the integrated gasification combined cycle procedure and the separation of H_2_ during ammonia production and methane reformation. The apparent activation energies of permeation for H_2_ and CO_2_ are 3.8 and 7.0 kJ mol^−1^, along with deduced diffusion activation energies of 8.8 and 38.0 kJ^−1^, confirming a more active diffusion process for H_2_ than that for CO_2_. Under a humidity of 60% in the feed gas mixture of H_2_/CO_2_, the permeability of H_2_ and CO_2_ and H_2_/CO_2_ selectivity display insignificant fluctuation due to a limited water molecule adsorption ability at relatively low humidity. Beyond its humidity stability, Zn–P-dmbIm liquid also has the capability to repair cracks when it is melted. This unique property allows the membrane to restore its structural integrity after being subjected to bending stress (Fig. 18F), despite Young's modulus of 13.1 GPa indicating susceptibility to fragility upon bending, as determined through nanoindentation.

**Fig. 19:**
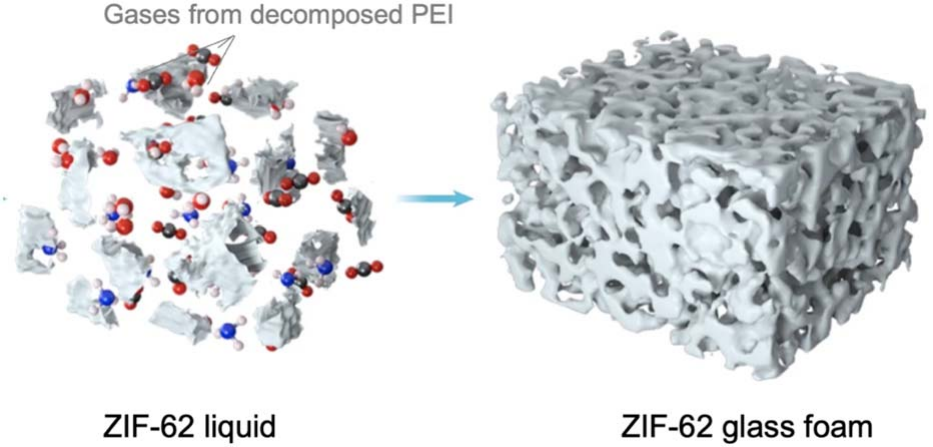
Formation mechanism of ZIF-62 glass foam. The PEI decomposition to CO_2_, NH_3_, and H_2_O increases pore connectivity in the glass membrane. Adapted with permission from ref. 140. Copyright 2023 Springer Nature Limited.

Differences in thermal stability are beneficial for creating extra pore networks within a self-supported glass monolith (glass foam, Fig. 19).^140^ A mixture of low-molecular-weight polyethylene-imine (PEI; *M*_w_ 300) and microcrystalline ZIF-62 was pressed and heated to 440 °C under an inert atmosphere. Thermal decomposition of PEI (160 to 370 °C) upon heating generates gases, including CO_2_, NH_3_, and H_2_O. These gases were then released upon cooling the ZIF-62 melts, thus introducing a more interconnected microporosity. This results in a membrane with higher porosity and a faster gas diffusion kinetic compared to the conventional ZIF-62 glass. The method provides circular membranes with a 3.3 cm diameter and a thickness between 200 and 330 μm that were tested for CH_4_/N_2_ separation. A high CH_4_ permeance of 30 000–50 000 GPU and permeability of *ca.* 10^7^ barrer, together with a high CH_4_/N_2_ selectivity of 4–6, were achieved. The values here contrast with the conventional ZIF-62 glass, as the CH_4_ permeance and CH_4_/N_2_ selectivity are 36.3 GPU and 1.1 due to the lack of pore connectivity.

### 4.3 Optical properties

The photophysical properties of CPs/MOFs vary between their crystalline and amorphous phases, and control over phase transition behavior is desirable for optics applications. This was exemplified in a series of isomeric copper CPs [Cu_4_I_4_L(MeCN)_2_] sol (L = *N*,*N*′-bis[2-(cyclohexylthio)-ethyl]pyromellitic diimide; sol = CH_2_Cl_2_, CHCl_3_, 0.5*p*-xylene, or nothing).^141^ The as-synthesized, crystalline CPs exhibit photoluminescence arising from the interaction between the Cu_4_I_4_ cluster and the ligand, with emission maxima in the 583–604 nm range. When subjected to heat-induced amorphization, the luminescence of the material is lost. When the amorphous CP is subsequently recrystallized through exposure to acetonitrile vapor, the emission is once again observed. This illustrates a promising application in vapochromic sensing.

**Fig. 20:**
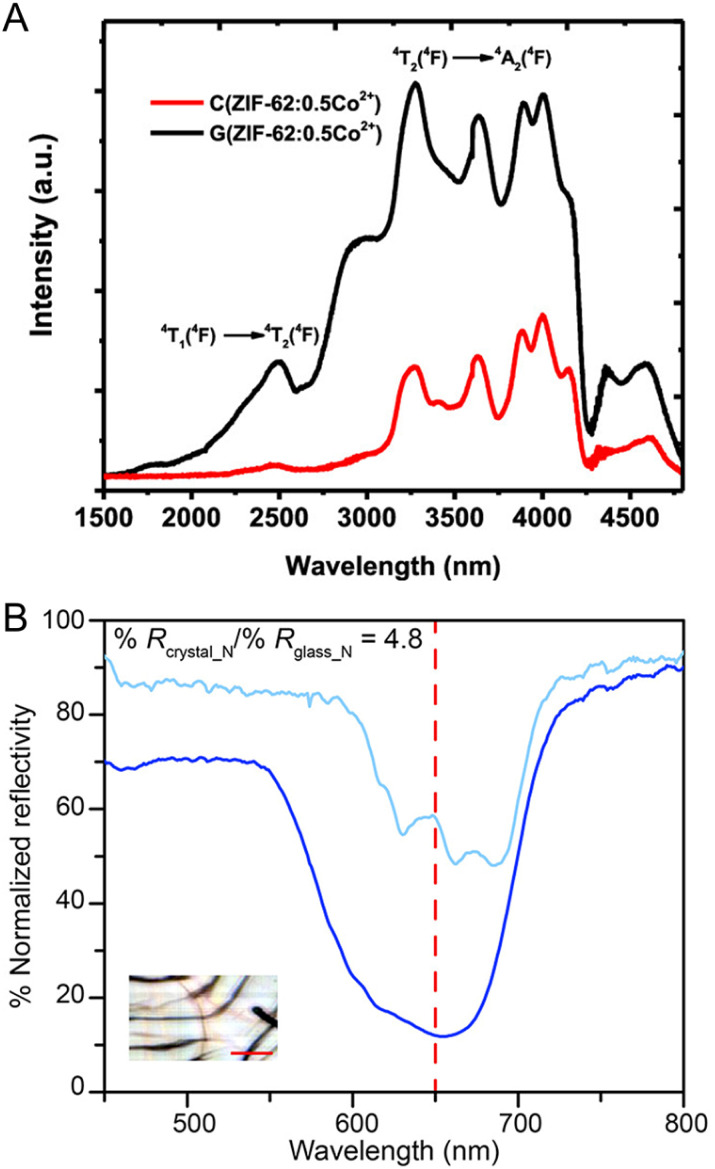
(A) PL spectra of the crystalline ZIF-62:0.5Co^2+^ and glass ZIF-62:0.5Co^2+^ under excitation by 980 nm laser diodes. (B) High reflectivity contrast ratio of 4.8 at 650 nm (red dotted line) across a melt quench-annealing cycle of a 1-Mn/Co(Zn) film. The % normalized reflectivity of the glass and crystalline film is shown in dark and light blue solid lines, respectively. Inset: optical images of the measured crystalline film, from which a single grain with a width of ∼15 μm was selected for data analysis. The scale bar corresponds to 10 μm. (A) reprinted with permission from ref. 142. Copyright 2019 American Chemical Society under Creative Commons license. (B) reprinted with permission from ref. 44. Copyright 2022 American Chemical Society.

Melt-state processing is effectively used in the design of optical devices. Co^2+^-doped ZIF-62 exhibits broad emission in the mid-IR region (*λ*_em_ = 1.5–4.8 μm), and photoluminescence enhancement occurs upon formation of the melt-quenched glass (Fig. 20A).^142^ While the native Zn–ZIF-62 does not show any absorption or emission in the visible to mid-IR regions, doping with Co^2+^ (10% or 50%) gave rise to absorption bands at 570 and 1100 nm, consistent with Co^2+^ in a CoN_4_ tetrahedral environment as it would be bound by imidazole and benzimidazole. The subsequent photoluminescence (*λ*_ex_ = 980 nm) was assigned to the ^4^T_1_(^4^F) → ^4^T_2_(^4^F) and ^4^T_2_(^4^F) → ^4^A_2_(^4^F) transitions of the Co^2+^ center, with increasing intensity correlating with increased Co^2+^ doping. While the photoluminescence intensity of the glassy state was greater than that of the crystalline state, this is mainly attributed to differences in sample morphology and scattering effects. The short-range structural order of ZIF-62 glass has been shown to stay intact upon melt-quenching, so the environment around the emitting Co^2+^ center is expected to be similar in both crystal and glass. This highlights the versatility of CP/MOF glasses, wherein the environment around the metal center is more or less retained.

Towards applications in information science, another series of metal-bis(acetamide) frameworks was synthesized, this time opting for the shorter *N*,*N*′-ethylenebis(acetamide) (eba) in M′(eba)_3_[M′′Cl_4_] compounds (M′ = M′′ = Mn^2+^, Fe^2+^, and M′ = Mn^2+^, Fe^2+^, Co^2+^, M′′ = Zn^2+^).^44^ They were expected to have a higher *T*_g_ than CPs constructed from the longer hmba or bba linkers, increasing their workable temperature range. Changes in optical properties upon phase transition (*i.e.*, crystal to melt-quenched glass) were studied in pure samples and binary mixtures formed by mixing parent compounds in the liquid state, followed by quenching to glass and thermal annealing to crystallize the mixture (Fig. 20B). The mixture of Mn(eba)_3_[ZnCl_4_] and Co(eba)_3_[ZnCl_4_] (1-Mn/Co(Zn)) was found to have the most pronounced differences between crystalline and glassy states, with the CP glass exhibiting a 5.4 times greater absorption coefficient (*α*) and the crystalline state having 4.8 times greater normalized reflectivity, both measured at 650 nm. The distinct optical differences in 1-Mn/Co(Zn) glassy and crystalline states were attributed to different Co^2+^ coordination environments. The faint blue absorption in crystalline 1-Mn/Co(Zn) is attributed to d–d transitions of [CoCl_4_]^2−^ tetrahedra, while transitions of the [CoO_6_]^2+^ octahedra are forbidden by symmetry. *In situ* formation of [CoCl_4_]^2−^ was further confirmed by EXAFS, as it was not present in either of the starting CPs. The formation of the glass lowers the average coordination number of [CoO_6_]^2+^ centers, breaking local symmetry and subsequently allowing the previously forbidden d–d transitions, resulting in a deep blue glass.

### 4.4 Thermal energy storage

Thermal energy is stored in materials in various forms, including thermochemical, sensible, and latent heat. Among these, latent heat storage stands out for its relatively high energy density and the added advantage of its isothermal nature.^143^ This concept aligns with the reversible phase transformation observed in metal–organic hybrid materials, where the transition between crystalline solid and disordered liquid states plays a significant role. Furthermore, strategic ligand design and precise coordination bonds enable control over factors like dimensionality, entropy, and the strength of intermolecular and intramolecular interactions. This control, in turn, leads to predictable thermodynamic and kinetic properties of phase transitions.^144^

The potential for manipulating order-to-disorder phase transitions in metal–organic compounds to achieve thermal energy storage has been showcased in a series of isostructural metal–organic coordination complexes labeled as [M(L)_6_]X_2_ (M = Mg^2+^, Ca^2+^, Mn^2+^, Co^2+^, Ni^2+^, Cu^2+^, Zn^2+^; L = *N*-methylurea (MeUr) or acetamide (AcNH_2_) or AcNH_2_ with MeOH; X = Cl^−^ or NO_3_^−^).^89^ Pairing metal halides with nitrate salts forms a series of octahedral complexes with directional intramolecular hydrogen bonds. These compounds show melting at 76.2–190.9 °C, and the melting enthalpy (Δ*H*_fus_) ranges from 155 to 357 kJ L^−1^ depending on the choices of metal ions and ligands. The overall enthalpic change during the transition is primarily influenced by the density and strength of both hydrogen and coordination bonds, which are highly dependent on the identity of metal cations and the orientation of counter-anions. Notable alterations within the first coordination sphere during melting also signify Δ*S*_fus_, and thus Δ*H*_fus_, upon transition. This is because of the increased rotational, vibrational, and translational degrees of freedom that become accessible to the dissociated ligands. Apart from melting behaviors, other types of phase transitions, such as conformation changes in organic constituents^145^ or spin state transitions of metal ions,^146^ have the potential to be useful for storing and releasing thermal energy.

## 5. Perspective

Similar to the properties of crystals, the properties of CP/MOF liquids (melting point, viscosity, structure, *etc.*) are correlated with the combination of metal ions and ligands and the structural dimensionality. A comparison between the metal–ligand bond strengths of different materials to gain additional understanding of these melting behaviors will provide an important guideline for designing new meltable CPs/MOFs.

**Fig. 21:**
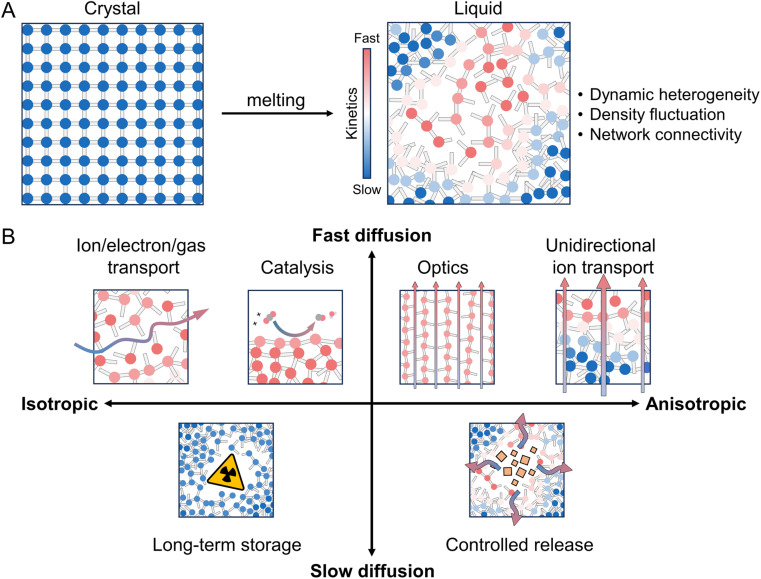
Schematic illustrations of future ideas. (A) Control of structural dynamics in liquid states. (B) Potential applications of CPs/MOFs in their liquid state based on their isotropic/anisotropic natures and kinetics.

One of the fundamental challenges is controlling the dynamic structure inherent in CP/MOF liquids (Fig. 21A). There are questions: does it take on a network nature connected by coordination bonds—on what timescale do the cleavage, reformation, and exchange of coordination bonds take place—are there phenomena such as dynamic heterogeneity?

A better understanding of these fundamentals will open up possibilities for material applications (Fig. 21B). One is the transport function using the liquid state. The targets are small molecules, including gases, ions, and even electrons. Separation by selective gas transport would be possible.^124^ If they show fast and selective ion transport, they are used in a variety of electrochemical devices. If a gradient of liquid structures is created, it could potentially give rise to anisotropic properties. An example here is unidirectional ion transport.^147^ Designing electrically conductive liquids is more difficult, but the local coordination and assembled structures can be controlled in a similar way to electrically conductive glasses.^148^ They could be platforms for sensors, dielectrics, energy storage, *etc.* Liquids also have the advantage of forming a mutual interface with different substances. Ionic liquids play an important role as electrolytes in iontronics due to their high capacitance and carrier surface density.^149–151^ Liquid metal catalysts are also attracting attention,^152^ and CP/MOF liquids may also play a role in such applications. In addition to transport, dissolution and decomposition in liquids are also important functions. For example, highly active, unstable species and substances, such as radioactive and volatile materials, can be dissolved and stored. Conversely, the decomposition of stable substances in the medium may also be possible.

Another challenge is the control of phase transitions. In liquid–solid (crystals and glasses) and even liquid–gas transitions, parameters such as operating temperature, heat balance, volume change, domain size, and response time can be studied for applications. Reversible and fast liquid–solid transitions are used to develop functions for heat and data storage. Unstable materials are encapsulated in a liquid medium, and upon solidification (vitrification or crystallization) upon cooling, both protective functions and transparency can be achieved. Control of the energy landscape also allows the creation of semi-melted (semi-crystalline) phases often found in conventional organic polymers. In other words, by designing domain structures in which the supercooled liquid phase and the crystalline phase coexist in the material, dual properties of tough mechanics and physical properties are expected. In addition to the liquid and solid phases, if the gas phase can be introduced in the future, large-area crystal films and giant single crystals can be produced by dry processes such as CVD and ALD. The liquid chemistry and phase transitions of CP/MOFs with different structural order, composition, and dynamics have great potential in various aspects, including energy harvesting, resource recycling, and scalability, as well as the fundamental development of novel disordered systems.

## Author contributions

The manuscript was written with the contributions of all authors. All authors have approved the final version of the manuscript.

## Conflicts of interest

There are no conflicts to declare.

## Supplementary Material
